# Microglia determine an immune-challenged environment and facilitate ibuprofen action in human retinal organoids

**DOI:** 10.1186/s12974-025-03366-x

**Published:** 2025-04-03

**Authors:** Verena Schmied, Medina Korkut-Demirbaş, Alessandro Venturino, Juan Pablo Maya-Arteaga, Sandra Siegert

**Affiliations:** Institute of Science and Technology Austria (ISTA), Am Campus 1, 3400 Klosterneuburg, Austria

**Keywords:** Human induced pluripotent stem cells, Retinal organoid, Microglia, Prenatal, Neuro-immune challenge, POLY(I:C), Ibuprofen, Prostaglandin, COX1, PTGS1, TORCH

## Abstract

Prenatal immune challenges pose significant risks to human embryonic brain and eye development. However, our knowledge about the safe usage of anti-inflammatory drugs during pregnancy is still limited. While human induced pluripotent stem cells (hIPSC)-derived brain organoid models have started to explore functional consequences upon viral stimulation, these models commonly lack microglia, which are susceptible to and promote inflammation. Furthermore, microglia are actively involved in neuronal development. Here, we generate hIPSC-derived microglia precursor cells and assemble them into retinal organoids. Once the outer plexiform layer forms, these hIPSC-derived microglia (iMG) fully integrate into the retinal organoids. Since the ganglion cell survival declines by this time in 3D-retinal organoids, we adapted the model into 2D and identify that the improved ganglion cell number significantly decreases only with iMG presence. In parallel, we applied the immunostimulant POLY(I:C) to mimic a fetal viral infection. While POLY(I:C) exposure alters the iMG phenotype, it does not hinder their interaction with ganglion cells. Furthermore, iMG significantly enhance the supernatant’s inflammatory secretome and increase retinal cell proliferation. Simultaneous exposure with the non-steroidal anti-inflammatory drug (NSAID) ibuprofen dampens POLY(I:C)-mediated changes of the iMG phenotype and ameliorates cell proliferation. Remarkably, while POLY(I:C) disrupts neuronal calcium dynamics independent of iMG, ibuprofen rescues this effect only if iMG are present. Mechanistically, ibuprofen targets the enzymes cyclooxygenase 1 and 2 (*COX1/PTGS1* and *COX2/PTGS2*) simultaneously, from which iMG mainly express *COX1*. Selective *COX1* blockage fails to restore the calcium peak amplitude upon POLY(I:C) stimulation, suggesting ibuprofen’s beneficial effect depends on the presence and interplay of *COX1* and *COX2*. These findings underscore the importance of microglia in the context of prenatal immune challenges and provide insight into the mechanisms by which ibuprofen exerts its protective effects during embryonic development.

**Supplementary Information:**

The online version contains supplementary material available at 10.1186/s12974-025-03366-x.

## Introduction

Prenatal exposure to infections can be detrimental to human embryonic development [1, 2]. Certain infectious diseases like rubella belonging to the TORCH complex (Toxoplasmosis, Others, Rubella, Cytomegalovirus, Herpes) can be vertically transmitted from pregnant women to their fetus, resulting in malformations of the fetal brain and eye [3–6]. Medication is recommended to a certain degree to treat inflammatory symptoms during pregnancy, but there are significant knowledge gaps on the effects of anti-inflammatory drugs on embryonic development [7].

Brain organoids derived from human induced pluripotent stem cells (hIPSCs) provide a unique strategy to investigate the consequences of prenatal inflammation, which we refer to as neuro-immune challenge, and drug exposure to this environment. Specifically, retinal organoids are one of the first established brain region-specific models [8]. Their developmental trajectories and cytoarchitecture are well-defined [9, 10] and match anatomical observations in human fetal retinal development, like the formation of the ganglion cell layer and the outer plexiform layer (OPL) [11]. At the same time, neuroectodermal-derived organoids commonly lack mesodermal-derived brain-resident macrophages [12], which colonize the human fetal brain and eye between gestation week (GW) 4.5 and 5 [13, 14]. Once in the neuronal environment, these microglia have multifunctional developmental tasks demonstrated in the rodent nervous system. They regulate, amongst others, the number of neural precursor cells [15, 16], axonal outgrowth and neuronal wiring [17], as well as synaptogenesis and pruning [18, 19] across various brain regions [20–23]. Microglia maintenance and survival depend on the colony-stimulating factor 1 receptor (CSF1R) [24–26], which, when inhibited, affects the total number of neurons and the macroglia cell populations consisting of astrocytes, oligodendrocytes and also their migration, distribution, and the functional connectivity [17, 21, 27–30]. Embryonic death and brain malformation have been reported in humans harboring homozygous mutations within the CSF1R genome [31, 32].

In recent years, protocols have been developed to generate hIPSC-derived microglia precursor cells (preMG), which acquire microglia-like cell (iMG) properties once integrated into neuroectodermal tissue and exposed to the environmental cues [33, 33–37, 37, 38]. Recent studies demonstrate that iMG promote brain organoid maturation [38] and fine-tune their neuronal environment at the cellular and synaptic levels [39–41]. Thus, microglia integration seems relevant to mimic in vivo human brain development.

Human cerebral organoids have been used to model the consequences of TORCH viruses such as Zika, and a reduction in neuronal progenitor numbers [42–46]. However, due to the lack of microglia in these studies, our insights into the inflammatory response and its consequences on human embryonic development are limited. Microglia are susceptible to environmental cues beyond pathogens [47], including inflammatory mediators such as cytokines and chemokines [48–50]. In rodent models, prenatal neuro-immune challenges induce microglia to express receptors to sense pathogens and inflammatory mediators [51] and affect microglia properties such as morphology, motility, and their actual number [16, 17, 52–54]. In parallel, these immune challenges also affect neurogenesis [15, 16, 20], neuronal differentiation [55], synaptogenesis [56, 57], and synaptic pruning [58, 59], to which microglia regularly contribute. Although microglia are critical to sense and adopt a response against infectious agents [48] their impact on the neuronal organization and connectivity in an inflammatory environment and the consequences of an anti-inflammatory treatment are poorly understood.

Here, we mimicked a prenatal neuro-immune challenged environment and subsequent treatment with the non-steroidal anti-inflammatory drug (NSAID) ibuprofen in microglia-assembled retinal organoids (iMG-_3D_RO). After we identified the optimal time-point to investigate microglia-neuron interaction in the hIPSC-derived 3D-retinal organoid (_3D_RO) [60], we developed a 2D-model system (_diss_RO) that mimics the retinal development the closest [60] and counteracts the previously reported ganglion cell loss in _3D_RO [10, 61, 62]. This model also circumvents known challenges of organoid-to-organoid variability in size, shape, and cell type composition [10, 63, 64], as well as diffusion biases of drugs. In this iMG-_diss_RO model, iMG actively interact with and phagocytose retinal ganglion cells as anticipated [10, 61, 62].

We then modeled a prenatal neuro-immune challenge by exposing the culture to the immunostimulant *polyinosinic: polycytidylic acid* (POLY(I:C)), which mimics a viral-mediated response and activates the toll-like receptor 3 (TLR3) [65]. TLR3 stimulation induces a downstream signaling cascade involving NFkB- and interferon pathways, resulting in cytokines and chemokines release [47, 66, 67]. Furthermore, POLY(I:C) directly acts on microglia as they upregulate *TLR3* mRNA expression [68]. We investigated the consequences of POLY(I:C)-mediated immune challenge in our iMG-_diss_RO model and identified a microglia-dependent inflammatory signature and increased retinal cell proliferation. To evaluate the effects of anti-inflammatory drugs on the identified consequences, we focused on the NSAID ibuprofen, which can be taken cautiously during the first half of the pregnancy [69]. Ibuprofen targets cyclooxygenase 1 and 2 (*PTGS1*/COX1, *PTGS2*/COX2, respectively) and prevents arachidonic acid conversion into prostaglandins like PGE2 [70, 71]. In the presence of ibuprofen, POLY(I:C)-mediated effects on microglia were dampened, and the neuronal phenotypes were restored. Yet, this beneficial effect depended on *PTGS1* expressed by iMG since ibuprofen did not show this rescue in cultures without iMG.

Our study highlights the interplay of human microglia with neurons during normal development, under prenatal neuro-immune challenges, and after anti-inflammatory drug exposure. Across all three conditions, we identified microglia-dependent phenotypes, emphasizing their significance. In light of future clinical drug tests in organoid models and known species-specific differences in microglial gene signature associated with immune response and neurodegenerative diseases [72, 73], microglia contribution cannot be excluded from experiments.

## Results

### OPL formation aligns with successful iMG integration into retinal organoids

**Fig. 1:**
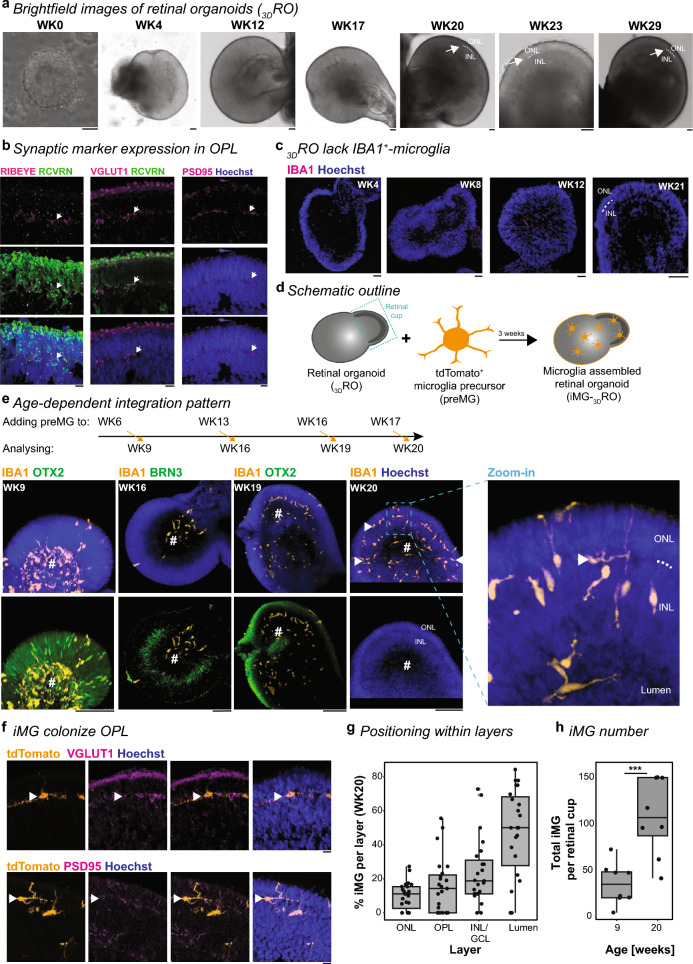
Microglia colonize retinal layers after OPL formation. **a** Representative brightfield images focusing on the retinal cup at selected _3D_RO differentiation time points. Arrow and dashed line: outer plexiform layer formation, visible from WK20 onwards. Scale bar: 100 µm. **b**, **c** Images of _3D_RO cryostat sections counterstained with the nuclei-dye Hoechst (blue) and immunostained for **b** photoreceptors with RCVRN (green) and the ribbon synapse marker RIBEYE (magenta, left); presynaptic marker VGLUT1 (magenta, middle); and postsynaptic marker PSD95 (magenta, right) at WK20. White arrow: outer plexiform layer. Scale bar: 10 µm. **c** IBA1 (magenta) at WK4, WK8, WK12, and WK21. Scale bar: 100 µm. **d** Experimental schematic to generate iMG-_3D_RO. **e** Maximum intensity projection image of entire iMG-_3D_RO counterstained with the nuclei-dye Hoechst (blue) at different time points of preMG application and collection of iMG-_3D_RO as outlined in the schematic. Immunostaining for IBA1 (orange), BRN3 (green, for WK16), and OTX2 (green, for WK9 and WK19). White arrowhead: iMG positioned in OPL. White dashed line: OPL. #: retinal cup lumen. Scale bar: 100 µm. Zoom-in: 10 µm. **f** Images of iMG-_3D_RO cryostat sections with tdTomato^+^-iMG (orange) at WK20 immunostained for the presynaptic marker VGLUT1 (magenta, top) or the postsynaptic marker PSD95 (magenta, bottom). Blue: nuclei-dye Hoechst. White arrowhead: iMG located in OPL. Scale bar: 10 µm. **g**, **h** Boxplot. **g** Percent of iMG in the ONL, OPL, INL, GCL, and within the retinal cup lumen (#) at WK20. Each dot: one cryostat section of an independent retinal cup. **h** Total number of iMG integrated per retinal cup at WK9 and WK20. Each dot represents an entire retinal cup. Students’s t-test. *** p < 0.001. For detailed statistical analysis, see Supplementary Table 4. *BRN3* brain-specific homeobox/POU domain protein 3B, *GCL* ganglion cell layer, *hIPSC* human induced pluripotent stem cell, *IBA1* ionized calcium-binding adapter molecule 1 (alternative name: AIF1), *INL* inner nuclear layer, *iMG* microglia-like cell, *iMG-*_*3D*_*RO* microglia assembled 3D-retinal organoid, *ONL* outer nuclear layer, *OPL* outer plexiform layer, *OTX2* orthodenticle homeobox 2, *preMG* microglia precursor cells, *PSD95* postsynaptic density protein 95, *RCVRN* recoverin, _*3D*_*RO* 3D-retinal organoid, *VGLUT1* vesicular glutamate transporter 1, *WK* week after the start of _3D_RO differentiation

To generate retinal organoids (_3D_RO), we differentiated the human induced pluripotent stem cell (hIPSC) line F49B7, which has been recently analyzed for its transcriptional cell diversity across different time points of _3D_RO differentiation [10]. We monitored the retinal cup formation under brightfield microscopy over 30 weeks (Fig. 1a) and observed the formation of the outer plexiform layer (OPL) around week (WK) 20. When we performed immunostaining for the presynaptic markers VGLUT1 and the post-synaptic marker PSD95 at WK13, 17, and 20, the staining was confined to the OPL at WK20 (Supplementary Fig. 1a, b). By WK20, we also found the presynaptic marker RIBEYE limited to the OPL (Fig. 1b). Furthermore, we confirmed the existence and the location of the different cell types within their expected nuclear layer at WK20, such as RCVRN^+^-/OTX2^+^-/CALB2^+^-photoreceptors in the outer nuclear layer and OTX2^+^/CALB2^+^-bipolar cells, CALB2^+^-amacrine cells, CALB1^+^-horizontal- and amacrine cells, and CHAT^+^-amacrine cells in the inner nuclear layer (Supplementary Fig. 1c). Few BRN3^+^-ganglion cells localized close to the _3D_RO lumen. RLBP1^+^-Müller glia cells expanded their processes across all layers. OPL formation and cell type expression patterns matched the anticipated timeline observed in human fetal tissue studies [74–76].

Human microglia have been shown to accumulate at the optic disc between GW10-13 and then populate the OPL between GW20-25 [60]. When we stained _3D_RO for the microglia-associated marker IBA1 [77], we did not find innately developing IBA1^+^-microglia within the retinal cup at any collected time points (Fig. 1c). This is in line with our previous observations [34] and confirms the sequencing data at weeks 30 and 38 by Cowan et al*.* which failed to identify microglia signature gene transcripts like IBA1/AIF1, CX3CR1, PU.1/SPI1, and P2RY12 (Supplementary Fig. 1d) [78–81]. Therefore, we focused on a microglia-assembled retinal organoid (iMG-_3D_RO) model, for which we developed a hIPSC line expressing the red fluorescent protein from the AAVS1 locus (Supplementary Fig. 2a–c) [82]. First, we confirmed that the hIPSC line remained pluripotent (Supplementary Fig. 2d) and successfully differentiated into tdTomato^+^/IBA1^+^-microglia precursor cells (preMG) expressing the previously described and expected preMG-markers [34] (Supplementary Fig. 2e–k). Then, we added tdTomato^+^-preMG to _3D_ROs at WK 6, 13, 16, or 17 of _3D_RO differentiation and followed their integration (Fig. 1d, e). Independent of the differentiation week of the organoid, tdTomato^+^-preMG attached to the developing outer nuclear layer within 24 h (Supplementary Fig. 3a). After a few days, the initially roundly-shaped preMG infiltrated into the _3D_RO and adapted their morphology into a bipolar profile, which spanned throughout the layers projecting towards the lumen of the retinal cup (Supplementary Fig. 3b). Differences in the preMG integration pattern correlated with the OPL formation. Before WK20, iMG preferentially accumulated in the lumen close to BRN3^+^-ganglion cells and rarely interacted with the developing retinal cells (Fig. 1e). After WK20, iMG integrated into the OPL (Fig. 1f, Supplementary Fig. 3c), or they extended their processes toward it (Fig. 1e). The position of the iMG soma indicated a spatial distribution across all retinal layers (Fig. 1g), and the total number of iMG significantly increased from WK9 to WK20 (Fig. 1h). Overall, we determined WK20 as the time point, which aligns with the microglia integration and spatial distribution pattern in human retinal development [60].

### iMG control ganglion cell number in adapted 2D-RO model with improved ganglion cell survival

**Fig. 2:**
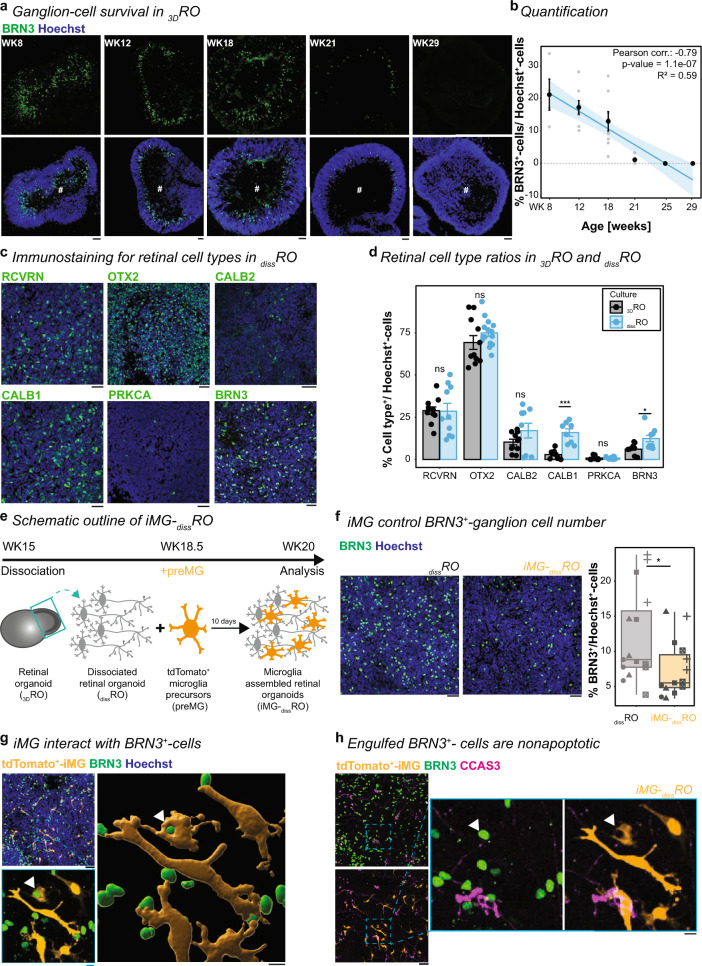
iMG interact with BRN3^+^-ganglion cells in dissociated retinal organoids. **a**, **b** Gradual ganglion cell loss with _3D_RO maturation. **a** Images of _3D_RO cryostat sections counterstained with the nuclei-dye Hoechst (blue) collected at WK8, 12, 18, 21, 29 and immunostaining for BRN3 (green). Scale bar: 50 µm. **b** Scatterplot of BRN3^+^-cells relative to Hoechst^+^-cells per cryostat section with SEM and trend curve. Pearson correlation with a significant negative correlation between the differentiation age and the BRN3^+^-cells number. **c**, **d** Retinal cell types in _diss_RO at WK20. Retinal cell type markers: RCVRN for photoreceptors, OTX2 for photoreceptors and bipolar cells, CALB2 for photoreceptors, bipolar- and amacrine cells, CALB1 for amacrine-, horizontal cells, PRKCA for rod bipolar cells, and BRN3 for ganglion cells. **c** Immunostaining for retinal markers (green) and nuclei-dye Hoechst (blue). Scale bar: 50 µm. **d** Bar chart with SEM of retinal cell types relative to Hoechst^+^-cells in _3D_ROs (black) and _diss_RO (blue). Each dot: cryostat section of individual _3D_ROs (black) or a field of view in _diss_RO (blue). Student’s t-test except for CALB1 (Wilcoxon rank-sum test). **e** Experimental timeline to generate iMG-_diss_RO. At WK15, retinal cups dissociated and plated as _diss_RO. At WK18.5, independently differentiated tdTomato^+^-preMG were added. iMG-_diss_RO was analyzed 10 days later at WK20. **f** Image of _diss_RO (left) and iMG-_diss_RO (right) at WK20 counterstained with the nuclei-dye Hoechst (blue) and immunostained for BRN3 (green). Scale bar: 50 µm. Next, boxplot of BRN3^+^-ganglion cells relative to Hoechst^+^-cells in _diss_RO (grey) and iMG-_diss_RO (orange). Symbols: single ROI of three biological replicates from five independent differentiation. Wilcoxon rank-sum test. **g**, **h** Representative images of BRN3^+^-ganglion cells (green), tdTomato^+^-iMG (orange), the nuclei-dye Hoechst (blue, **g**), and the apoptotic marker CCAS3 (magenta, **h**) of iMG-_diss_RO at WK20. Scale bar: 50 µm, zoom-in: 10 µm. Zoom-in: **g** 3D-surface rendering of a region of interest. White arrowhead: iMG engulfing BRN3^+^-cell. **h** White arrowhead: iMG engulfing BRN3^+^/CCAS3^−^-cell. For detailed statistical analysis, see Supplementary Table 4. ***p < 0.001. *p < 0.05. ^ns^p > 0.05, not significant. *BRN3* brain-specific homeobox/POU domain protein 3B, *CALB1* calbindin, *CALB2* calretinin, *CCAS3* cleaved caspase-3, *iMG-*_*diss*_*RO* microglia-assembled dissociated retinal organoids, *iMG-*_*3D*_*RO* microglia assembled 3D-retinal organoid, *iMG* microglia-like cells, preMG microglia precursor, *PRKCA* protein kinase C alpha, *OTX2* orthodenticle homeobox 2, _*3D*_*RO* 3D-retinal organoid, _*diss*_*RO* dissociated retinal organoid cultures without iMG, *RCVRN* recoverin, *ROI* region of interest, *SEM* standard error of the mean, *WK* week after the start of _3D_RO differentiation

In human fetal tissue, the ganglion cell layer fully forms by GW24 [11], and its formation is accompanied by extensive cell loss peaking between GW16 and 21 [83]. Microglia have been shown to interact with newborn BRN3^+^-ganglion cells and reduce their density in the rodent retina [21]. To recapitulate this phenotype in human _3D_RO is challenging due to the gradual loss of retinal ganglion cells with increasing maturation (Fig. 2a, b), a well-documented phenotype [10, 61, 62]. Therefore, we adapted recent protocols that dissociate 3D organoids, plated them as 2D cultures, and validated cortical network activity reestablishment [84, 85]. Dissociated retinal organoid culture (_diss_RO) will allow us to minimize diffusion biases and compare treatment paradigms directly because the wells derive from the same pool of dissociated WK15 _3D_ROs, circumventing organoid-to-organoid variability. Until WK20, retinal cells will have had sufficient time to successfully reform their synaptic connections [86]. First, we compared the cell type composition and density to the age-matched _3D_ROs (Fig. 2c, d). We found that the percentage of each cell type was similar between _3D_RO and _diss_RO with the exceptions of CALB1 and BRN3, which both significantly increased in _diss_RO (Fig. 2d). Importantly, brain-derived neurotrophic factor (BDNF) in the culture medium supported BRN3^+^-ganglion cell survival in _diss_RO compared to _3D_RO (Supplementary Fig. 4a).

Next, we added tdTomato^+^-preMG to _diss_RO at WK18.5 (iMG-_diss_RO, Fig. 2e). After 10 days in culture, iMG were distributed across the plate, representing 2.61% ± 1.13 of the total Hoechst^+^-nuclei number (Supplementary Fig. 4b). To evaluate if the co-culture promotes iMG maturation (Supplementary Fig. 4c) [35, 36, 38, 78, 87], we performed RT-qPCR of iMG-_diss_RO. On day 1, we observed the mRNA expression of microglia-associated markers such as C1QA, CX3CR1, P2RY12, and TMEM119 (Supplementary Fig. 4c) [78, 80], which were not detected in the absence of iMG (Supplementary Fig. 1d). Except for TMEM119, their mRNA expression significantly increased over 9 days, indicating that co-culturing with _diss_RO promoted microglia marker expression on the mRNA level. On day 10, iMG expressed the transcription factors for primitive macrophage development PU.1 and RUNX1, and MYB was no longer present (Supplementary Fig. 4e) [88]. iMG also expressed IBA1, CD45 as well as more mature microglia marker TREM2 and P2RY12 (Supplementary Fig. 4f, g).

To understand the functional consequences of iMG integration, we investigated their phagocytic ability. Similar to rodent studies [21], the number of BRN3^+^-ganglion cells significantly reduced in iMG-_diss_RO compared to _diss_RO (Fig. 2f). Furthermore, 22.53% ± 7.13% of all BRN3^+^-ganglion cells positioned within a 5 μm radius of iMG. An average of 1.72 ± 1.70 iMG engulfed BRN3^+^-ganglion cell bodies (Fig. 2g), indicating their role in regulating neuron number during development. To determine if these engulfed cells are apoptotic ganglion cells, we co-labeled the culture with the apoptotic marker cleaved caspase-3 (CCAS3). CCAS3^+^-cells were present (Fig. 2h), but the total number was unaffected by iMG presence (Supplementary Fig. 5a). In line with previous observations in rodents [21], iMG engulfed non-apoptotic ganglion cells (CCAS3^−^/BRN3^+^) (Fig. 2h). At WK20, iMG selectively targeted ganglion cells as all phagocytosed Hoechst^+^-nuclei co-expressed BRN3^+^. On a note, the number of Hoechst^+^-cells or OTX2^+^-photoreceptor/ bipolar cells remained unaffected at WK20 (Supplementary Fig. 5b, c).

On a side note, the CCAS3 staining also highlighted that iMG removed cellular debris exemplified in their processes surrounding Hoechst^+^/CCAS3^+^-nuclear fragments (Supplementary Fig. 5d). Consequently, iMG-_diss_RO contained fewer Hoechst^+^-nuclear fragments than cultures without iMG (Supplementary Fig. 5e, f). Also, in _3D_ROs, iMG phagocytosed Hoechst^+^-nuclear fragments (Supplementary Fig. 5 g), emphasizing their phagocytic role in both models [20].

### POLY(I:C) affects iMG phenotype without interfering with the ganglion cell interaction

**Fig. 3:**
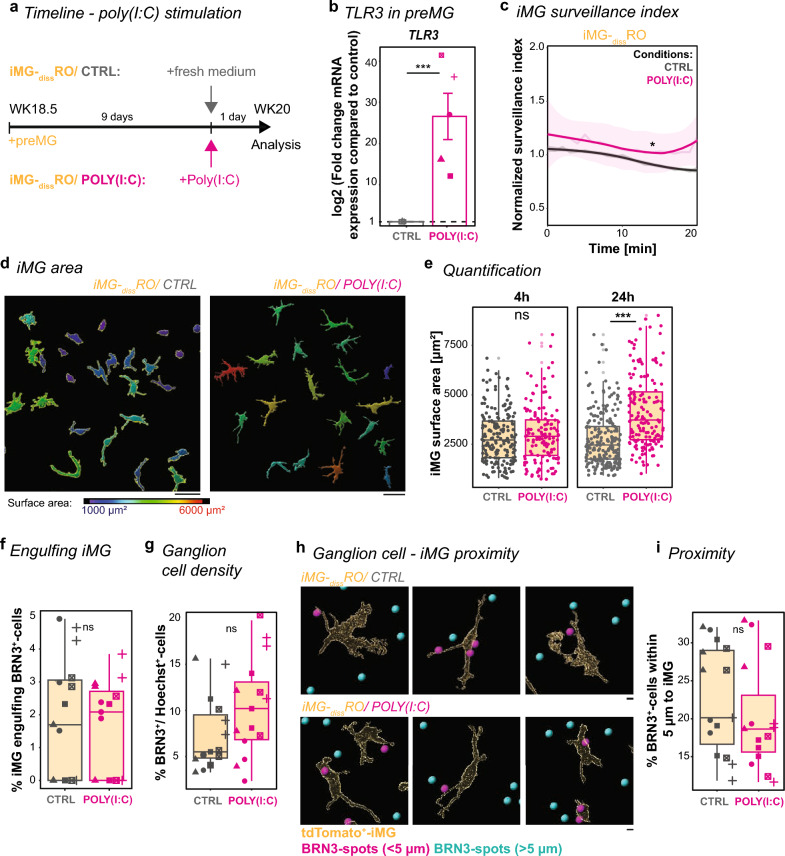
iMG respond to POLY(I:C) stimulation but still interact with ganglion cells. **a** Experimental timeline. At WK18.5, preMG are added to _diss_RO. After 9 days, the culture medium was replaced with fresh medium either containing POLY(I:C) (magenta) or without as a control (CTRL, grey). Analysis was performed 24 h later on day 10. **b** RT-qPCR for TLR3 of preMG after CTRL or POLY(I:C) stimulation. Bar chart with SEM: Mean mRNA transcript log2-fold changes compared to CTRL. Symbol: mean of technical triplicate from five independent differentiations. One sample t-test. **c** iMG-_diss_RO live imaging for 20 min for CTRL or POLY(I:C) stimulation after 24 h. iMG surveillance index normalized to the mean surveillance of the cells in CTRL with a 95% confidence interval. Four independent differentiations. Wilcoxon rank-sum test. **d**, **e** iMG surface area quantification.** d** iMG surface rendering for CTRL (left) and POLY(I:C) (right) 4 h and 24 h following stimulation, color-coded based on surface area: blue = 1000 µm^2^ to red = 6000 µm^2^. Scale bar: 50 µm. **e**, Boxplot of individual iMG surface areas in iMG-_diss_RO for CTRL and POLY(I:C). iMG were collected from five independent differentiations. Kruskal–Wallis test with post-hoc Dunn’s test. **f**, Boxplot quantifying the number of iMG engulfing BRN3^+^-cells in iMG-_diss_RO for CTRL and POLY(I:C). Symbols: single ROI of three biological replicates from five independent differentiations. Wilcoxon rank-sum test. **g** Boxplot determines ganglion cell density based on BRN3^+^-cells relative to Hoechst^+^-cells in iMG-_diss_RO for CTRL and POLY(I:C). Symbols: single ROI of three biological replicates from five independent differentiations. Wilcoxon rank-sum test. **h**, **i** Ganglion cell-iMG proximity in iMG-_diss_RO. **h** Surface rendering of iMG for CTRL (top) and POLY(I:C) (bottom). BRN3^+^-spots color-coded based on the proximity to the iMG surface with spots < 5 µm (magenta) and spots > 5 µm (cyan). Scale bar: 10 µm. **i** Boxplot of percent of magenta BRN3^+^-spots. Symbols: single ROI of three biological replicates from five independent differentiations. Wilcoxon rank-sum test. For detailed statistical analysis, see Supplementary Table 4. ***p < 0.001. * p < 0.05. ^ns^p > 0.05, not significant. BRN3: brain-specific homeobox/POU domain protein 3B. *CTRL* untreated control, *iMG* microglia-like cells, *iMG-*_*diss*_*RO* microglia integrated into dissociated retinal organoid culture, *POLY(I:C)* polyinosinic: polycytidylic acid, *preMG* microglia precursor cells, _*diss*_*RO* dissociated retinal organoid cultures, *RT-qPCR* real-time quantitative polymerase chain reaction, *ROI* region of interest, *SEM* standard error of the mean, *TLR3* toll-like receptor 3, *WK* week after the start of _3D_RO differentiation

To mimic a prenatal neuro-immune challenge in our WK20 culture, we applied POLY(I:C) for 24 h (Fig. 3a). This immunostimulant activates a TLR3 response cascade, triggering downstream signaling pathways related to immune defense [89, 90]. Indeed, *TLR3* mRNA level significantly increased after POLY(I:C) stimulation in preMG culture (Fig. 3b), supporting a direct effect of POLY(I:C) on iMG. Next, we monitored iMG activity for 20 min in iMG-_diss_RO (Supplementary videos 1, 2). We found that iMG surveillance significantly increased compared to the control condition without POLY(I:C) (Fig. 3c). Furthermore, iMG significantly enlarged their surface area at 24 h post-stimulation (Fig. 3d, e), and they showed changes in other morphological features commonly found in reactive microglia [91, 92].

Based on these iMG phenotypes, we revisited the previously observed iMG-ganglion cell interaction (Fig. 2f, g). iMG engulfed a comparable number of ganglion cells to age-matched, untreated control conditions (Fig. 3f). When we analyzed the number of BRN3^+^-ganglion cells, we observed a trend towards an increase in POLY(I:C)-treated culture, but this effect was insignificant (Fig. 3g). We thus also investigated whether the iMG interaction with BRN3^+^-ganglion cells is altered and determined the iMG position within a 5 μm radius of BRN3^+^-labeling. The proximity measurement did not reveal an apparent difference between POLY(I:C) -stimulated and non-stimulated conditions (Fig. 3h, i), suggesting that POLY(I:C) does not have an immediate effect on the iMG developmental task to regulate the ganglion cell number.

### iMG presence influences POLY(I:C)-mediated inflammatory secretome signature and cell proliferation

**Fig. 4:**
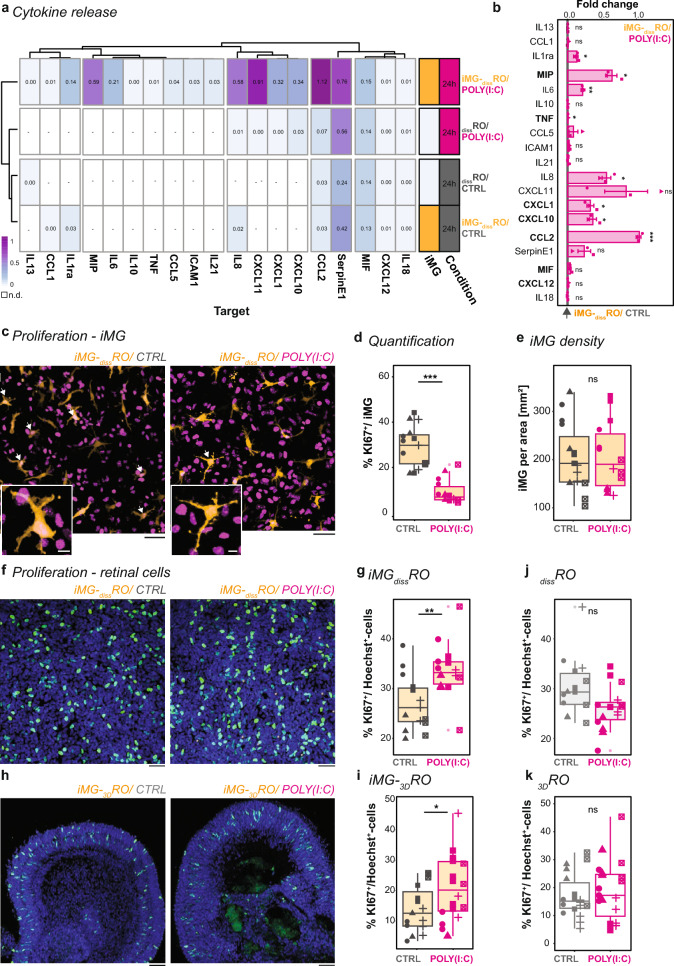
POLY(I:C)-mediated microglia-dependent consequences on the retinal environment. **a**, **b** Release of inflammatory cytokines and chemokines into the supernatant based on the experimental paradigm described in Fig. 3a for control (CTRL, grey) and POLY(I:C) (magenta) after 24 h stimulation. **a** Heatmap with color-coded mean pixel intensity relative to the reference of three independent differentiations (for individual plots, see Supplementary Fig. 5a). White: n.d. Side-bar: condition with iMG (orange) or without (white) or CTRL *versus* POLY(I:C). **b** Bar chart with SEM: Fold change of pixel intensity upon POLY(I:C) stimulation relative to CTRL. Each dot is an independent differentiation (n = 3). Shapiro–Wilk normality test < 0.05, Wilcoxon rank-sum test. Shapiro–Wilk normality test > 0.05, one sample t-test. **c**–**e** iMG proliferation rate in iMG-_diss_RO for CTRL (left) and POLY(I:C) (right). **c** Example ROI image of tdTomato^+^-iMG (orange) and immunostained for the proliferation marker KI67 (magenta). White arrow: KI67^+^-expressing iMG. Scale bar: 50 µm. Zoom-in: Scale bar: 10 µm. **d** Boxplot of KI67^+^/iMG percentage. Wilcoxon rank-sum test. **e** Boxplot of iMG per area. Students’ t-test. **d**, **e** Symbols: single ROI of three biological replicates from five independent differentiations. **f**–**k** Proliferation of retinal cells for iMG-_diss_RO (**f**, **g**), iMG-_3D_RO (**h**, **i**), _diss_RO (**j**), and _3D_RO (**k**). Example ROI images counterstained for the nuclei-dye Hoechst (blue) and immunostained for the proliferation marker KI67 (green) for CTRL (left) and POLY(I:C) stimulation (right) for iMG-_diss_RO (**f**) and cryostat section with a focus on the retinal cup of iMG-_3D_RO (**h**). Scale bar: 50 µm. Boxplot percent of KI67^+^-cells relative to Hoechst^+^-cells, excluding KI67^+^/iMG, for CTRL and POLY(I:C) in iMG-_diss_RO (**g**), iMG-_3D_RO (**i**), _diss_RO (**j**), and _3D_RO (**k**). Symbols: single ROI of three biological replicates from five independent differentiations. Students’ t-test. For detailed statistical analysis, see Supplementary Table 4. ***p < 0.001. **p < 0.01. *p < 0.05. ^ns^p > 0.05, not significant. *CTRL* untreated control, *iMG* microglia-like cells, *iMG-*_*diss*_*RO* microglia integrated into dissociated retinal organoid culture, *iMG-*_*3D*_*RO* microglia assembled 3D-retinal organoids, *KI67* marker of proliferation KI-67, *n.d*. not detectable, *POLY(I:C)* polyinosinic: polycytidylic acid, _*diss*_*RO* dissociated retinal organoid cultures without iMG, *ROI* region of interest, *SEM* standard error of the mean, *WK* week after the start of _3D_RO differentiation

To obtain insights into how iMG presence affects the POLY(I:C)-mediated neuro-immune response, we analyzed the supernatant of _diss_RO and iMG-_diss_RO after 24 h of POLY(I:C) stimulation and compared it to the untreated control (Fig. 4a).

At baseline without POLY(I:C) stimulation, _diss_RO with or without iMG were comparable, showing a similar set of secreted mediators, including MIF, CCL2, CXCL12, IL18, and SerpinE1 (Fig. 4a, Supplementary Fig. 6a, b). _diss_RO exposed to POLY(I:C) formed a separate cluster with only moderate differences from the controls. The additional detected cytokines CXCL10, CXCL11, CXCL1, and IL8 belong to the CXC family and are known to be secreted by astrocytes [93–95]. Indeed, we verified the presence of GFAP^+^-glia cells in _diss_RO (Supplementary Fig. 6c). The most robust inflammatory secretome signature occurred when we stimulated iMG-_diss_RO with POLY(I:C). The previous four factors were significantly higher released, and we detected an additional eight secreted inflammatory mediators such as TNFα, IL6, and MIP (Fig. 4a, b). Since those factors have already been partially upregulated in _diss_RO, iMG seemed to amplify the signal. On a note, approximately half of the inflammatory mediators assayed were not secreted in any condition (Supplementary Fig. 6d), and also not after only 2- or 4 h of POLY(I:C) exposure in iMG-_diss_RO (Supplementary Fig. 7a). In contrast, iMG morphology started already to adapt 4 h post-stimulation (Fig. 3c, d, Supplementary Fig. 7b), suggesting that iMG activation occurs before the release of inflammatory mediators.

CCL2 (C–C Motif Chemokine Ligand 2) has been one of the strongest upregulated factors upon POLY(I:C) stimulation in iMG-_diss_RO (Fig. 4b). Besides being involved in the homing of monocytes and T-cells from the periphery [96, 97], CCL2 also contributes to neuronal proliferation in concert with the other upregulated secreted factors CXCL12, MIF, MIP, TNFα, CXCL1, and CXCL10 [28, 30, 98–101] (Fig. 4b). To investigate the consequences on the number of proliferating cells, we immunostained for the proliferation marker KI67 (Fig. 4c–k). In iMG-_diss_RO, POLY(I:C) stimulation significantly reduced the number of KI67^+^/iMG-cells compared to the control (Fig. 4c, d), which supports data in rodents [102]. Yet, the overall iMG density remained similar (Fig. 4e), suggesting that iMG are less proliferative. In contrast, the overall number of proliferating retinal cells excluding iMG significantly increased upon POLY(I:C) stimulation in iMG-_diss_RO (Fig. 4f, g). We observed the same effect in iMG-_3D_ROs (Fig. 4h, i) emphasizing that this effect is independent from _diss_RO versus _3D_RO. Overall, this consequence aligns with rodent studies after prenatal immune challenges [16, 20, 103]. Since the secretion of proliferation-associated factors was only upregulated in the presence of iMG (Fig. 4a), we determined the number of KI67^+^/Hoechst^+^-cell in _diss_RO and _3D_ROs lacking iMG. POLY(I:C) failed to increase KI67^+^-cells in both conditions (Fig. 4j, k), emphasizing an iMG-dependent effect on cell proliferation upon POLY(I:C) exposure.

Next, to identify whether CCL2 is the primary mediator of this effect, we applied 10 ng/ml CCL2 to iMG-_diss_RO cultures at WK20 and analyzed the consequences 24 h later (Supplementary Fig. 8a). In contrast to POLY(I:C) stimulation, CCL2 exposure did not increase the overall proliferation rate (Supplementary Fig. 8b). Even if we applied higher CCL2 concentrations, the ratio of KI67^+^/Hoechst^+^-cells remained the same. Unexpectedly, the ratio of KI67^+^/iMG rose with 10 ng/ml CCL2 (Supplementary Fig. 8c), which is in contrast to the POLY(I:C) condition (Fig. 4c, d). This suggests that CCL2 alone cannot drive the observed phenotypes and that the interplay with additional proliferation-associated factors is critical.

### Ibuprofen dampens POLY(I:C)-induced iMG phenotypes and reduces cell proliferation

**Fig. 5:**
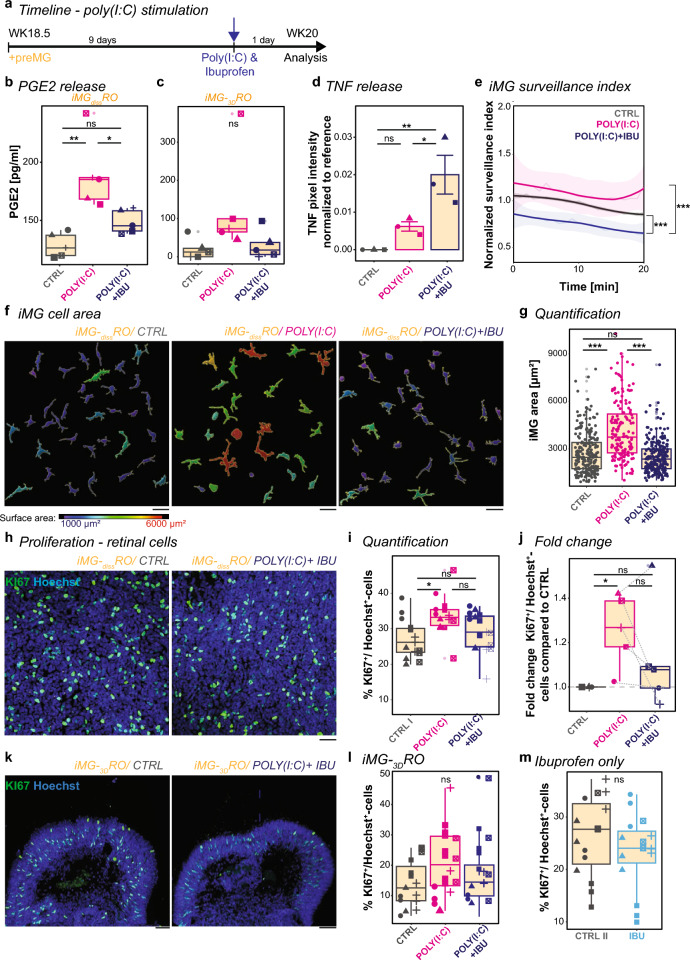
Ibuprofen partially reverses POLY(I:C)-mediated consequences on iMG and cell proliferation. **a** Experimental timeline. At WK18.5, preMG are added to _diss_RO. After 9 days, cultures received fresh medium for control (CTRL, grey), POLY(I:C) (magenta), or POLY(I:C) and S(+)-ibuprofen (POLY(I:C) + IBU, blue) for 24 h before analysis. **b**, **c** PGE2 is released into the supernatant of iMG-_diss_RO (**b**) and iMG-_3D_RO (**c**). Boxplot of PGE2 concentration [pg/ml] after CTRL, POLY(I:C), and POLY(I:C) + IBU. Each symbol: an independent differentiation (n = 5). **b** One-way ANOVA with post-hoc Tukey’s test. **c** Kruskal–Wallis test with post-hoc Dunn’s test. **d** Release of TNF into the supernatant of iMG-_diss_RO. Boxplot of pixel intensity normalized to reference for CTRL, POLY(I:C), and POLY(I:C) + IBU. Each symbol is an independent differentiation (n = 3). One-way ANOVA with post-hoc Tukey’s test. **e** iMG-_diss_RO live imaging for 20 min after 24 h stimulation. iMG surveillance index normalized to the mean surveillance of the cells in CTRL with a 95% confidence interval. Data from five independent differentiations. Kruskal–Wallis test with post-hoc Dunn’s test. **f**, **g** iMG surface area in iMG-_diss_RO. **f** iMG surface rendering for CTRL (left), POLY(I:C) (middle), and POLY(I:C) + IBU (right), color-coded based on surface area: blue = 1000 µm^2^ to red = 6000 µm^2^. Scale bar: 50 µm. **g** Boxplot of individual iMG surface areas. iMG from five independent differentiations. Kruskal–Wallis test with post-hoc Dunn’s test. **h**–**m**, Proliferation rate of retinal cells in iMG-_diss_RO (**h**–**j**, **m**) and iMG-_3D_RO (**k**, **l**). **h** and **k**, Example ROI of iMG-_diss_RO (**h**) and cryostat sections focusing on retinal cup iMG-_3D_RO (**k**) at WK20 counterstained with the nuclei-dye Hoechst (blue) and immunostained for KI67 (green) for CTRL (left) and POLY(I:C) + IBU (right). Scale bar: 50 µm. **i**, **j** and **l**, **m**, Boxplot percent of KI67^+^-cells relative to Hoechst^+^-cells for CTRL, POLY(I:C), and POLY(I:C) + IBU excluding KI67^+^/iMG. Each symbol is an independent differentiation (n = 5). Single ROI of three biological replicates. **i** One-way ANOVA with post-hoc Tukey’s test. **j** Fold change of median percent of KI67^+^-cells relative to Hoechst^+^-cells compared to CTRL. One sample t-test and Student’s t-test. **l** Kruskal–Wallis test. **m** Boxplot percent of KI67^+^-cells relative to iMG in iMG-_diss_RO for CTRL and only IBU exposure (light-blue) for 24 h. Students’s t-test. For detailed statistical analysis, see Supplementary Table 4. ***p < 0.001. **p < 0.01. *p < 0.05. ^ns^p > 0.05, not significant. *CTRL* untreated control, _*diss*_*RO* dissociated retinal organoid cultures without iMG, *IBU* S(+)-ibuprofen, *iMG* microglia-like cells, *iMG-*_*diss*_*RO* microglia integrated into dissociated retinal organoid culture, *iMG-*_*3D*_*RO* microglia assembled 3D-retinal organoids, *KI67* marker of proliferation KI-67, *PGE2* prostaglandin E2, *POLY(I:C)* polyinosinic: polycytidylic acid, *POLY(I:C) + IBU* POLY(I:C) and S(+)-ibuprofen, *preMG* microglia precursor cells, *ROI* region of interest, *TNF* tumor necrosis factor alpha, *WK* week after the start of _3D_RO differentiation

Besides cytokines and chemokines, another hallmark of inflammation is the secretion of prostaglandins such as PGE2, which mediate classic symptoms of inflammation [104, 105]. Indeed, we found that iMG-_diss_RO stimulated with POLY(I:C) for 24 h showed increased PGE2 levels in the supernatant (Fig. 5a, b). NSAIDs like ibuprofen target the enzymes cyclooxygenase 1 and 2 (*PTGS1*/COX1, *PTGS2*/COX2, respectively) and prevent arachidonic acid conversion into prostaglandins [70, 71]. Simultaneous exposure of POLY(I:C) with the active enantiomer S(+)-ibuprofen dampened PGE2 upregulation in iMG-_diss_RO (Fig. 5b) as well as in iMG-_3D_RO (Fig. 5c). Next, we investigate the release of cytokine and chemokine into the supernatant during the POLY(I:C)-mediated neuro-immune challenge when we simultaneously applied S(+)-ibuprofen. Most inflammatory mediators remained unaffected upon exposure to S(+)-ibuprofen (Supplementary Fig. 9a). Only TNF secretion increased (Fig. 5d), which aligns with a previous study identifying that PGE2 inhibits TNF expression in macrophage cell lines in vitro [106].

Since microglia have been shown to constitutively express PTGS1/COX1 [107] and ibuprofen targets PTGS1/COX1, we revisited iMG surveillance and monitored their activity (Supplementary Video 3). 24 h following exposure of S(+)-ibuprofen simultaneously with POLY(I:C), iMG surveillance significantly reduced compared to just POLY(I:C) and even below the control level in _diss_RO (Fig. 5e). Morphologically, iMG remained confined, exhibiting less cell surface area than just POLY(I:C) exposure (Fig. 5f, g), indicating a dampened activity and underlining a direct ibuprofen-mediated effect on iMG.

To further examine whether ibuprofen improves the consequences of the prenatal neuro-immune challenge, we revisited the increased proliferation phenotype upon POLY(I:C) exposure. Following simultaneous treatment with S(+)-ibuprofen, the ratio of KI67^+^/Hoechst^+^-cells reduced for four out of five differentiation in iMG-_diss_RO compared to POLY(I:C) stimulation alone (Fig. 5h–j). In iMG-_3D_RO, we observed a similar beneficial effect (Fig. 5k, l).

Since ibuprofen has been associated with anti-proliferative effects in cancer cell lines [108, 109], we evaluated S(+)-ibuprofen without POLY(I:C) exposure in iMG-_diss_RO. The number of proliferating cells remained similar (Fig. 5m).

### Ibuprofen depends on iMG to reverse POLY(I:C)-mediated effects on neuronal activity

**Fig. 6:**
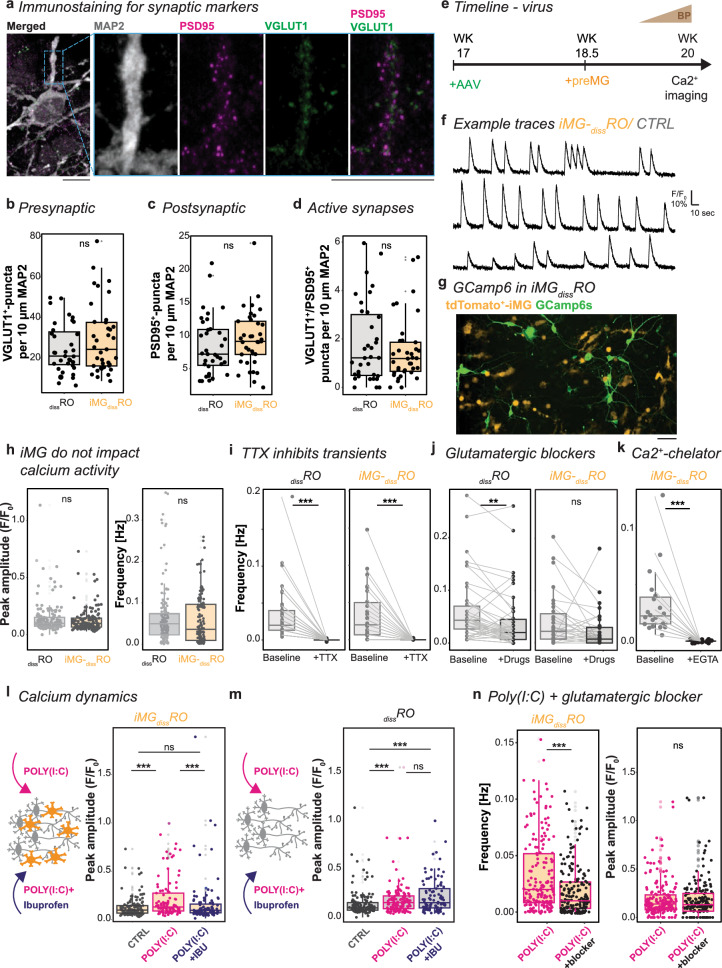
Calcium dynamics are affected upon POLY(I:C), yet ibuprofen needs iMG to reverse the phenotype. **a** Example image of iMG-_diss_RO immunostained for the neuronal marker MAP2 (grey), the presynaptic marker VGLUT1 (green), and the postsynaptic marker PSD95 (magenta) with zoom-in. Scale bar: 10 µm. **b**–**d** Boxplots of synaptic puncta quantification in _diss_RO (grey) and iMG-_diss_RO (orange) for VGLUT1^+^-puncta per 10 µm MAP2 (**a**), PSD95^+^-puncta per 10 µm MAP2 (**b**) and VGLUT1^+^/PSD95^+^-puncta per 10 µm MAP2 (**c**). Each dot represents one process close to the soma of a single cell. Five independent dissociations from three differentiations. Wilcox-test. **e** Experimental timeline. At WK17, _diss_RO transduced with AAV2-GCAMP6s. preMG added at WK18.5. Four days before calcium imaging, gradual transition to Brain-Phys medium until WK20. **f** Three example traces of spontaneous calcium transients in iMG-_diss_RO. **g** Example ROI image of iMG-_diss_RO expression of the calcium sensor GCAMP6s (green) and tdTomato^+^-iMG (orange). Scale bar: 50 µm. **h** Spontaneous calcium dynamics during 5 min recording in _diss_RO (grey) and iMG-_diss_RO (orange). Boxplot of the mean frequency [Hz] (left) and the mean peak amplitude [F/F_0_] (right). Wilcoxon rank-sum test. **i** TTX abolishes calcium transients in _diss_RO (left) and iMG-_diss_RO (right). Boxplot of mean frequency [Hz] during 150-s baseline recording (baseline) following TTX application for another 150 s (+ TTX). One-sample Wilcoxon signed rank test. **j** Boxplot of mean frequency [Hz] during 150-s baseline recording (baseline) following exposure to glutamatergic blockers CPP, APB, and NBQX for 150 s in _diss_RO (left) and iMG-_diss_RO (right). Wilcoxon rank-sum test. **k** Boxplot of mean frequency [Hz] during 150-s baseline recording (baseline) following Ca2^+^-chelator EGTA application for another 150 s in iMG-_diss_RO. One-sample Wilcoxon signed rank test. **l**, **m** Spontaneous calcium dynamics during 5 min recording after 24 h of either fresh medium for control (CTRL, grey), POLY(I:C) (magenta), or POLY(I:C) and S(+)-ibuprofen (POLY(I:C) + IBU, blue) exposure in iMG-_diss_RO (**i**) and _diss_RO (**j**). Boxplot of the mean peak amplitude [F/F_0_]. Each dot represents an active cell. Recordings from five independent differentiations. Kruskal–Wallis test with post-hoc Dunn’s test. **k** Spontaneous calcium dynamics in iMG-_diss_RO after 24 h POLY(I:C) stimulation and following exposure to glutamatergic blockers CPP, APB, and NBQX. Boxplot of the mean frequency [Hz] (left) and the mean peak amplitude [F/F_0_] (right). Each dot represents an active cell from five independent dissociations from three independent differentiations. Wilcoxon rank-sum test.For detailed statistical analysis, see Supplementary Table 4. ***p < 0.001. **p < 0.01. ^ns^p > 0.05, not significant. *AAV* adeno-associated virus, *APB* 2-aminoethoxydiphenyl borate, *CPP* 4-(3-phosphonopropyl)piperazine-2-carboxylic acid, *CTRL* untreated control, _*diss*_*RO* dissociated retinal organoid cultures without iMG, *EGTA* Ethylene glycol tetraacetic acid, *IBU* ibuprofen, *iMG* microglia-like cells, *iMG-*_*diss*_*RO* microglia integrated into dissociated retinal organoid culture, *MAP2* microtubule-associated protein 2, *NBQX* 2,3-dioxo-6-nitro-7-sulfamoyl-benzo[f]quinoxaline, *POLY(I:C)* polyinosinic: polycytidylic acid, *PSD95* postsynaptic density protein 95, _*diss*_*RO* dissociated retinal organoid cultures, *Sec* seconds, *TTX* tetrodotoxin, *VGLUT1* vesicular glutamate transporter 1, *WK* week after the start of _3D_RO differentiation

Initially, we described that the OPL formation aligned with iMG integration. Once the OPL is formed, spontaneous glutamatergic activity shapes neuronal circuits in vivo [110]. Since iMG-_diss_RO expressed synaptic markers (Fig. 6a), we investigated the number of presynaptic VGLUT1^+^-puncta and postsynaptic PSD95^+^-puncta on MAP2^+^-neurons, and their proximity as an indicator for functional synapses (VGLUT1^+^/PSD95^+^-puncta) in _diss_RO and iMG-_diss_RO. iMG presence affected neither of those parameters at WK20 (Fig. 6b–d).

Next, we visualized spontaneous calcium transients as a correlate for neuronal activity. First, we transduced _diss_RO with adeno-associated virus (AAV), which is independent of TLR3-signaling [111]. The AAV encoded for the calcium sensor GCAMP6s under the control of the ubiquitous EF1α promoter [10, 112], resulting in a broad expression across retinal cell types (Supplementary Fig. 9a–e)*.* Importantly, to exclude any AAV-mediated microglia activation, we applied the virus to _diss_RO at WK17 and 1.5 weeks before adding preMG (Fig. 6e). At WK20, we analyzed the spontaneous calcium transients (Fig. 6f, g, Supplementary Video 4). The calcium peak amplitude and the mean frequency remained similar in _diss_RO and iMG-_diss_RO (Fig. 6h). The calcium transients were either abolished after application of the voltage-gated sodium channel blocker tetrodotoxin (TTX) (Fig. 6i, Supplementary Video 5) or significantly reduced after pharmacological blocking of glutamatergic synaptic transmission using a combination of CPP, NBQX, and APB (Fig. 6j, Supplementary Video 6). Furthermore, the spontaneous calcium activity depended on extracellular calcium because the transients stopped when we applied the Ca^2+^-chelator EGTA into the media (Fig. 6k, Supplementary Video 7).

Inflammatory factors have been shown to modulate neuronal activity [113–116]. Indeed, we found that POLY(I:C) exposure in both iMG-_diss_RO and _diss_RO significantly increased the calcium peak amplitude of individual cells (Fig. 6l, m) and had no effect on the mean frequency (Supplementary Fig. 10f). To obtain insights into whether the increased calcium peak amplitude is mediated via glutamatergic synaptic transmission, we applied CPP, NBQX, and APB following 24 h POLY(I:C) stimulation. Similar to the untreated control (Fig. 6j), the frequency significantly decreased (Fig. 6n). We found that the amplitude was unaffected, suggesting that the increase in the calcium amplitude peak is independent of glutamatergic signaling and instead mediated through microglia-neuron signaling.

Finally, when we simultaneously applied POLY(I:C) and S(+)-ibuprofen, strikingly, the peak amplitude was only restored to the control condition in iMG-_diss_RO (Fig. 6l). In _diss_RO, the peak amplitude remained elevated compared to the control (Fig. 6m), and the frequency was unaltered (Supplementary Fig. 10f). This data suggests that iMG presence is critical for the effect of ibuprofen on restoring POLY(I:C)-induced changes in the calcium dynamics.

### Both ibuprofen targets, PTGS1 in microglia and PTGS2, are required to rescue calcium dynamics

**Fig. 7:**
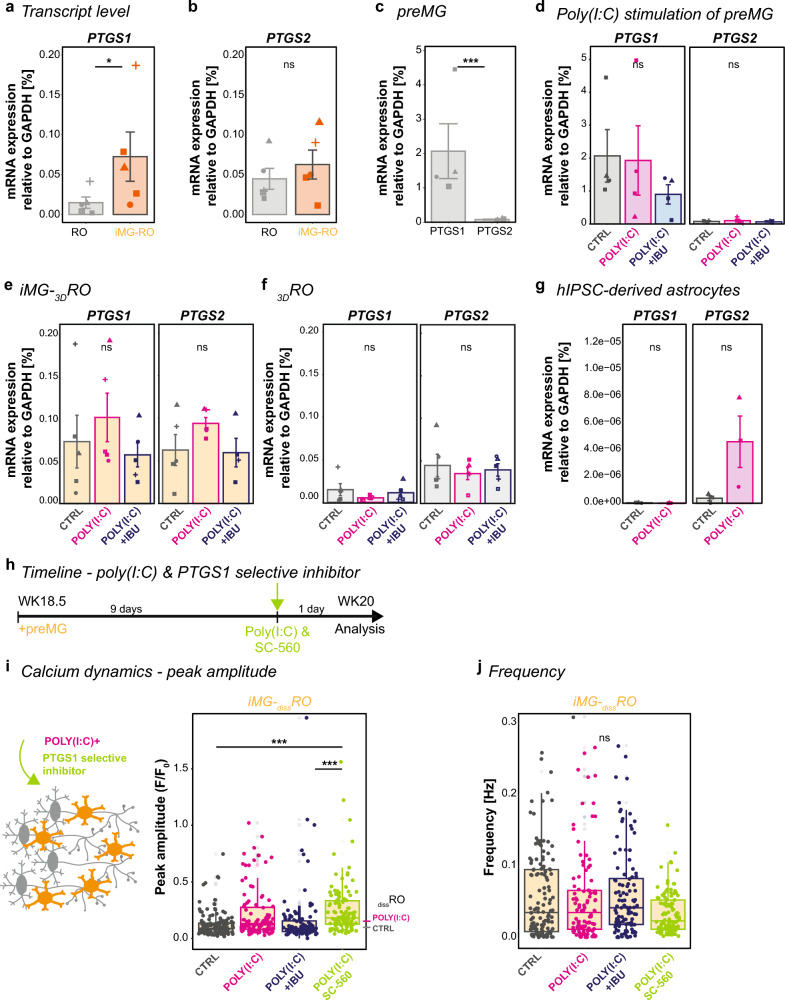
Cell type-dependent transcriptional differences in PTGS1 and PTGS2 point to iMG impact on calcium dynamics. **a**–**g** RT-qPCR. Bar chart with SEM of mean mRNA transcript expression relative to GAPDH for PTGS1 (**a**, **c**–**g**) and PTGS2 (**b**–**g**) across untreated (**a**–**c**) and CTRL *versus* POLY(I:C) (magenta) or POLY(I:C) (**g**) and S(+)-ibuprofen (POLY(I:C) + IBU, blue) (**d**–**f**) for _3D_RO *versus* iMG-_3D_RO (**a**, **b**), preMG (**c**, **d**), iMG-_3D_RO (**e**), _3D_RO (**f**) and hIPSC-derived astrocytes (**g**). **h** Experimental timeline similar to with POLY(I:C) and SC-560 exposure for 24 h in iMG-_diss_RO at WK20. Students t-test (**a**–**c**). One-way ANOVA (**d**–**g**) except g/PTGS2 Kruskal–Wallis test. **i**, **j** Spontaneous calcium dynamics in iMG-_diss_RO during 5 min recording after 24 h of either fresh medium for control (CTRL, grey), POLY(I:C) (magenta), POLY(I:C) and S(+)-ibuprofen (POLY(I:C) + IBU, blue), or POLY(I:C) and SC-560 (POLY(I:C) + SC-560, green) exposure. Boxplot of mean peak amplitude [F/F_0_, **i**] and mean frequency [Hz, **j**]. Each dot represents an active cell from five independent differentiations. Peak amplitude: reference lines for the median of the control and POLY(I:C) stimulation in _diss_RO from Fig. 6m. Kruskal–Wallis test with post-hoc Dunn’s test. For detailed statistical analysis, see Supplementary Table 4. ***p < 0.001. *p < 0.05. ^ns^p > 0.05, not significant. *CTRL* untreated control, *GAPDH* glyceraldehyde 3-phosphate dehydrogenase, *H* hour, *hIPSC* human induced pluripotent stem cell, *IBU* ibuprofen, *iMG* microglia-like cells, *iMG-*_*diss*_*RO* microglia integrated into dissociated retinal organoid culture, *iMG-*_*3D*_*RO* microglia assembled 3D-retinal organoids, *POLY(I:C)* polyinosinic: polycytidylic acid, *preMG* microglia precursor cell, *PTGS1* prostaglandin-endoperoxide synthase 1 (COX1), *PTGS2* prostaglandin-endoperoxide synthase 2, _*3D*_*RO* 3D-retinal organoids, *RT-qPCR* real-time quantitative polymerase chain reaction, *SC-560* PTGS1/COX-1 selective inhibitor, *SEM* standard error of the mean, *WK* week after the start of _3D_RO differentiation

To identify the mechanism behind the iMG-dependent response upon ibuprofen exposure, we revisited the two main targets of ibuprofen, *PTGS1* and *PTGS2*, at the transcriptional level. In the _3D_RO sequencing data [10], *PTGS2* transcripts occurred in Müller glial and horizontal cells, whereas *PTGS1* transcripts were absent (Supplementary Fig. 10a). Since this dataset lacks iMG, we collected _3D_RO with and without iMG around WK20 and analyzed the mRNA transcript levels of both enzymes. *PTGS1* relative to *GAPDH* was significantly increased in iMG-_3D_RO compared to _3D_RO (Fig. 7a), whereas the *PTGS2* levels remained similar (Fig. 7b).

Microglia have been shown to express PTGS1 constitutively [107], and a common assumption is that PTGS2 is upregulated during inflammatory conditions [117]. To validate these assumptions, we investigated the transcript level in preMG cultures. Indeed, the absolute expression of *PTGS1* compared to *GAPDH* was significantly higher than that of *PTGS2* (Fig. 7c). Yet, neither PTGS1 nor PTGS2 levels increased upon POLY(I:C) exposure following POLY(I:C) stimulation (Fig. 7d). At the same time, we observed a trend for *PTGS1* and *PTGS2* increase in iMG-_3D_RO (Fig. 7e) which is absent in _3D_RO (Fig. 7f).

When we analyzed the cytokine assay (Fig. 4a, b), we observed several secreted factors known to be released by astrocytes. In _3D_RO, astrocytes/Müller glia show a low expression level of TLR3 (Supplementary Fig. 10b). We confirmed this expression in hIPSC-derived astrocyte cultures [118], which further upregulate *TLR3* transcripts upon POLY(I:C) exposure (Supplementary Fig. 10c). When we profiled *PTGS1* and *PTGS2*, we found that hIPSC-derived astrocytes significantly increased *PTGS2* transcripts (Fig. 7g). This emphasizes that preMG and astrocytes differentially express the enzymes cyclooxygenase 1 and 2, respectively.

Since ibuprofen targets both enzymes, we were interested in whether applying POLY(I:C) in combination with S(+)-ibuprofen results in an iMG-dependent effect on *PTGS1* and *PTGS2* expression. While the treatment reduced *PTGS1* mRNA expression in preMG (Fig. 7d), it indicates a reversal of the POLY(I:C)-mediated increase to control levels in iMG-_3D_RO (Fig. 7e). In _3D_ROs, S(+)-ibuprofen showed no trend (Fig. 7f), suggesting that iMG are involved in a beneficial ibuprofen effect.

Ibuprofen did not rescue the calcium peak amplitude in the absence of iMG (Fig. 6m), a condition in which PTGS1 is underrepresented (Fig. 7a). Since S(+)-ibuprofen targets both enzymes simultaneously, we selectively blocked PTGS1 to understand if the beneficial effect depends on microglial PTGS1. To test this hypothesis, we took advantage of the inhibitor SC-560, which has a 700-fold selectivity for PTGS1 over PTGS2 [119, 120] and, therefore, will directly affect iMG in iMG-_diss_RO. When we applied SC-560 together with POLY(I:C) for 24 h (Fig. 7h) and measured the calcium dynamics in iMG-_diss_RO, the calcium peak amplitude was similarly upregulated as in _diss_RO following ibuprofen treatment (Fig. 7i). The frequency remained unaffected (Fig. 7j). This suggests that inhibition of functional PTGS1 or the simple lack of iMG prevents the positive effect of ibuprofen from restoring the calcium peak amplitude, highlighting a critical interplay between PTGS1-expressing iMG and PTGS2-mediated POLY(I:C) upregulation in other cells.

## Discussion

This study highlights the importance of including microglia in hIPSC-neuronal organoids to mimic fetal brain development and adequately model pathogen- and ibuprofen-mediated processes.

### Prenatal neuro-immune challenge

TORCH infection (Toxoplasmosis, Others, Rubella, Cytomegalovirus, Herpes) in a pregnant woman challenges her immune system and can severely threaten the fetus [2, 3]. Through vertical transmission, TORCH can directly harm the developing fetus, inducing growth restrictions or birth defects such as blindness [121]. For example, the Zika virus has been shown to target human fetal microglia [122] and to activate the innate immune receptor TLR3 [46, 123]. Thus, we mimicked the TLR3 signaling pathway using POLY(I:C) [65]. In vivo, the receptor is expressed predominantly in microglia but also in astrocytes, endothelial cells, and pericytes, while only minimally in neurons and neuronal progenitors [51]. We confirmed this expression pattern in preMG (Fig. 3b) and hIPSC-derived astrocytes (Supplementary Fig. 11c). Furthermore, we demonstrated that 24 h of POLY(I:C) exposure resulted in a robust iMG-dependent cytokine release (Fig. 4a, b), accompanied by enhanced iMG surveillance (Fig. 3c), elevated calcium peak amplitude in retinal cells (Fig. 6l, m), and increased retinal cell proliferation (Fig. 4f–j). Our observations in _diss_RO lacking iMG showed a trend of reduced proliferation (Fig. 4j). Overall, our observations align with rodent models. The proliferation rate remained unaffected if microglia were depleted and the pregnant females were exposed to an immune challenge during embryonic and early postnatal stages [20]. Contrarily, the proliferation increased in the presence of microglia [16, 20, 103], comparable to our data (Fig. 4f–j). Microglia-dependent effects have also been reported on the transcriptional level after prenatal neuro-immune challenges, such as IFNɣ stimulation of brain organoids [124] or 3 days of POLY(I:C) exposure on E15.5 rodent brain [51], emphasizing the microglia relevance in the inflammatory response and therefore, their integration into organoids should be standard procedure to capture developmental effects fully.

### The presence of iMG determines ibuprofen’s beneficial effect

The observed POLY(I:C)-mediated consequences raise the question of how to treat infectious diseases during pregnancy to avoid adverse pregnancy outcomes [125]. NSAIDs act on prostaglandin G/H synthase 1 and 2 (PTGS1/COX1 and PTGS2/ COX2), resulting in anti-inflammatory, antipyretic, and analgesic properties [126, 127]. We decided to use ibuprofen because Germany allows this medication during the first two trimesters of pregnancy [69]. Ibuprofen crosses the placental barrier and accesses the developing fetus [128, 129]. We found that by applying ibuprofen simultaneously with POLY(I:C), we could ameliorate the increased cell proliferation (Fig. 5h–l), restore the calcium peak amplitude (Fig. 6l), and dampen the POLY(I:C)-mediated microglial response (Fig. 5e–g). Surprisingly, ibuprofen did not reduce most of the secreted inflammatory mediators in iMG-_diss_RO (Supplementary Fig. 9a), possibly due to the lack of infiltrating immune cells to resolve the inflammatory response.

iMG constitutively express PTGS1 over PTGS2 (Fig. 7c) and the beneficial effect of ibuprofen depends on iMG. In iMG absence, ibuprofen mainly interferes with PTGS2 in _diss_RO, which was insufficient to rescue the calcium peak amplitude (Fig. 6m). On the other hand, calcium dynamics also did not recover when we selectively inhibited PTGS1 with SC-560 (Fig. 7i). Since *PTGS2* seems to be preferentially expressed in astrocytes (Fig. 7g) and iMG interact extensively with them, a potential astrocyte-microglia link exists and warrants follow-up studies.

### The effects of microglia in retinal organoids

Recently, three studies have been published focusing on iMG integration into retinal organoids [39–41]. Both Usui-Ouchi et al*.* and Chichagova et al*.* performed their integration and investigation on a timeline similar to our study and compared _3D_RO with and without iMG. Usui-Ouchi et al*.* confirmed iMG integration into the developing OPL (Fig. 1e, f, Supplementary Fig. 3c) and shows that their presence temporarily increases the pro-inflammatory factors *IL1B*, *TNF,* and *IL6* using qRT-PCR. Our cytokine release assay did not detect differences in their secretion between _diss_RO and iMG-_diss_RO in untreated condition (Fig. 4a, Supplementary Fig. 6a-b, d), instead only after POLY(I:C) stimulation, IL-6 and TNF were released in iMG-_diss_RO. We also performed qRT-PCR for these three factors and observed a similar mRNA upregulation as the authors describe. However, POLY(I:C) stimulation exceeded at least 500-fold (data not shown). This indicates that translation from mRNA to an actual release into the supernatant might be tightly controlled. Chichagova et al*.* mimicked bacterial infection with lipopolysaccharide (LPS) and confirmed an iMG-dependent secretome using a different assay. We can observe differences in the response to LPS and POLY(I:C) as they, e.g., don’t observe an effect on CCL2/MCP-1, one of our strongest affected chemokines (Fig. 4a). Due to the lack of control conditions, they might have missed changes due to iMG integration, as reported before by Usui-Ouchi et al. Finally, the study by Gao et al*.* compares mostly macrophages not integrated into a neuronal environment, which we named in our study preMG, after either LPS and POLY(I:C) stimulation. We consider this comparison suboptimal due to the known effects of LPS and POLY(I:C) on neuronal populations [16, 20, 103], also exemplified in the impact on the calcium peak amplitude (Fig. 6l, m). The authors suggest a similar reduction in BRN3^+^-cells in their D30-old iMG-_3D_RO, as we have seen (Fig. 2f). Yet, they do not show iMG-BRN3^+^-interaction (Fig. 2g) and only describe that they found a few microglia in the retina without insights into their positioning, making it challenging to derive further conclusions. In summary, the above studies are inconclusive regarding the ideal timing for studying microglia-neuron interaction, and our study closes this knowledge gap.

## Conclusion

Our study provides a baseline for the neurodevelopmental role of microglia, the cross-talk with their neuron-glia environment, and how prenatal neuro-immune activation and anti-inflammatory drug treatment are affected. In light of replacing in vivo models for drug screening and validation for FDA drug approval [130] with in vitro models [131], integrating all relevant cell types becomes critical to achieving drug efficiency in screenings. The limitation of these models to replicate adequately an inflammatory response will severely affect the information regarding the safety of medications during pregnancy for both the pregnant woman and the fetus and will raise serious public health concerns. Specifically, our model can serve as a platform for follow-up investigations on drug tests or the interplay between inflammation and microglia activation leading to neurological phenotypes in adulthood. For example, a rubella infection during pregnancy is one of the risk factors for developing schizophrenia [132].

## Limitation

This study focuses on the acute response to an early-life neuro-immune challenge and how microglia contribute to the observed consequences (Figs. 3, 4, 5, 6). We did not further investigate potential long-term effects caused by, e.g., the increased proliferation rate (Fig. 4f, g), which could result in an imbalance of retinal cell type populations changing the cytoarchitecture, or the elevated calcium peak amplitude (Fig. 6l) may lead to altered network activity. iMG might be able to sense and compensate for POLY(I:C)-mediated consequences over time. Also, a prolonged stimulation period might be interesting, as infections usually last longer than 24 h. Furthermore, stimulation before WK20 or later could also result in different responses because the timing of an immune challenge contributes to differences in the outcomes [133, 134]. Finally, future models must expand on the possibility of including the blood–brain barrier into the system. Identified factors such as MIP, CCL5, CXCL1, and CCL2 (Fig. 4a) are known candidates for homing monocytes and T-cells from the periphery [97, 135–138] and might be needed to downregulate the inflammatory signature once recruited.

## Material and methods

### Ethical approval

The ISTA Ethics Officer and Ethics Committee approved the usage of human induced pluripotent stem cells (hIPSC).

### Cell lines

We used two human induced pluripotent stem cell lines (hIPSC): SC 102A-1 GVO-SBI Human Fibroblast-derived (feeder-free) IPSC line (BioCat; hPSCreg.eu: SBLi006-A; in this study referred to SC102A) and the human fibroblast-derived IPSC line 01F49i-N-B7 (Renner lab [10], in this study referred to F49B7). For more details, see (Supplementary Table 1).

### Cell culture and human induced pluripotent stem cells

#### Matrigel coating

Matrigel (Corning® Matrigel® hESC-Qualified Matrix, *LDEV-Free, Corning, #354277) was used according to the manufacturer protocol with the following modifications: Matrigel aliquots were dissolved in ice-cold X-Vivo 10 chemically defined, serum-free hematopoietic cell medium (Lonza, #BE04-380Q). Dishes were coated for 1 h at room temperature.

#### Maintenance of human induced pluripotent stem cells (hIPSCs)

hIPSCs were maintained in mTeSR1 medium (STEMCELL Technologies, #85850) on Matrigel-(Corning, #354277) coated 6-well plates (Corning, #3516) cultured at 37ºC and 5% CO_2_ in a humidified incubator (BINDER C150). Before reaching 80% confluency, hIPSCs were passaged as small aggregates every 3–4 days using EDTA dissociation buffer composed of 0.5 M EDTA (ethylenediaminetetraacetic acid, K.D. Biomedical, #RGF 3130), 0.9 g (w/v) NaCl (Sigma, #5886) in PBS (phosphate buffered saline, calcium/magnesium-free, Invitrogen, #14190), sterile filtered and stored at 4 °C according to [139]. The ISTA Molecular Biology Facility regularly tested hIPSCs for mycoplasma using the Multiplex qPCR assay—16S DNAaccording to [140].

#### Freezing and thawing of hIPSCs

For freezing, hIPSCs were washed once with DPBS (Thermo Fisher Scientific, #14190250), incubated in EDTA dissociation buffer for 2.5 min, detached as small aggregates using mFReezer (STEMCELL Technologies, #05854), and frozen at − 80 °C. For long-term storage, hIPSCs aliquots were transferred to liquid nitrogen. For thawing, hIPSCs were removed from liquid nitrogen and quickly thawed in a bead bath at 37 °C. hIPSCs were transferred into a falcon tube containing mTesR1 medium. The cells were centrifuged (VWR, Mega Star 3.0R) at 200×*g* for 3 min, then resuspended in mTesR1 medium and transferred into one well of a Matrigel-coated 6-well plate.

#### Generation of tdTomato expressing hIPSC lines

To generate ubiquitous tdTomato expressing hIPSC lines, a reporter construct encoding tdTomato under the constitutive enhancer/β-actin (CAG) promoter (2xCHS4-CAG-tdTomato-SV40-2xCHS4, gift from the Knoblich lab [141]) was inserted into the safe-harbor AAVS1 locus. A CRISPR/CAS9 approach was used, as previously described [82]. For nucleofection, 80% confluent hIPSCs were dissociated into single-cell suspension using Accutase (Merck, #SCR005) treatment for 4 min. Cells were collected, centrifuged (VWR, Mega Star 3.0R) at 200×*g* for 3 min, and resuspended in mTeSR1 medium supplemented with 10 µM ROCK inhibitor (Y-27632, STEMCELL Technologies #72307). The Human Stem Cell NucleofectorTM Kit 1 (Lonza, #VPH-5012) was applied using 1 million hIPSCs, 3 µg donor plasmid DNA, and 1 ug CRISPR/CAS9 guideRNA (pXAT2 plasmid, Addgene: #80494). After nucleofection, hIPSCs were distributed on six wells of a Matrigel-coated 6-well plate. Colonies from single cells were grown for 5–6 days in mTeSR1 medium. Then, tdTomato expressing hIPSCs were isolated using fluorescent activated cell sorting (FACS, Sony, SH800SFP). Therefore, transfected hIPSCs were collected using Accutase treatment for 4 min, centrifuged at 200*g* for 3 min, and resuspended in mTesR1 medium supplemented with 10 µM ROCK inhibitor. Using a 100 µm nozzle, 10.000 hIPSCs were sorted and distributed on three wells of a Matrigel-coated 6-well plate. After 4–5 days, 20 to 30 colonies, which were well separated from one another and evenly expressed tdTomato, were identified using an EVOS imaging system (Thermo Fisher Scientific). Identified colonies were manually picked with a 200 µL tip, transferred into a Matrigel-coated 96-well plate, and cultured in mTesR1 medium. The colonies were expanded into 24- (Corning, #3527), 12- (Corning, #3512), and 6-well plates (Corning, #3516). For passaging, refer to “*Maintenance of human induced pluripotent stem cells (hIPSCs)”.*

#### Validation of tdTomato expressing hIPSC lines

Half of the hIPSCs were collected for genotyping when splitting colonies from a 24-well plate to a 12-well plate. DNA was extracted using the DNeasy Blood and Tissue kit (QIAGEN, #69504). All reactions were performed using Q5 Hot Start High Fidelity 2× Master Mix (NEB, #M0494S) with 50–100 ng of template DNA per reaction. PCR was performed using the following primers to identify whether the insertion was heterozygous or homozygous: AAVS1_FWD: 5ʹ-TCGACTTCCCCTCTTCCGATG-3ʹ, AAVS1_WT_REV 5ʹ-CTCAGGTTCTGGGAGAGGGTAG-3ʹ and AAVS1_Insert_REV 5ʹ-GAGCCTAGGGCCGGGATTCTC-3ʹ as described previously [82]. The size of PCR products was analyzed by gel electrophoresis (wildtype allele 1.4 kbp and target allele 1.2 kbp). Clones with correctly targeted homozygous insertions were expanded.

### Differentiation of retinal organoids, astrocytes, and microglia precursor cells

#### Retinal organoid differentiation

3D-retinal organoids were generated as described with the following modifications [9, 10]: on day 0 of the differentiation, colonies of the hIPSC line F49B7 from two wells of a 6-well plate were cut into evenly sized aggregates using a cell-passaging tool (Thermo Fisher Scientific, #23181-010). After detaching, floating aggregates were transferred with a 1250 µL wide orifice pipette (VWR, #613-0737) into one 9-cm Petri dish (Sarstedt, #82.1473), and cultured in mTeSR1 medium supplemented with 10 µM blebbistatin (Sigma, #B0560-5MG). On day 1, 2, and 3, the medium was gradually exchanged with ¼, ½, and 1, respectively, to NIM (neural induction medium: DMEM/F12 (Gibco, #31331-028), 1× N2-supplement (Gibco, #17502-48), 1% (v/v) NEAA Solution (Sigma, #M7145), 2 µg/mL heparin (Sigma, #H3149-50KU)). From day 4 onwards, 10 mL NIM was changed daily. On day 8, embryoid bodies (EB) were equally distributed onto 8 Matrigel-coated 6-cm dish plates (Corning, #3516) (approximately 20–40 number of EBs/cm^2^) and cultured in 3 mL NIM. From day 16 onwards, NIM was replaced to 3:1-medium consisting of 3 parts DMEM (Thermo Fisher Scientific, #31966047) and one-part F12 medium (Ham’s F-12 Nutrient Mix, Thermo Fisher Scientific, #31765-027) supplemented with 2% (v/v) B27 without vitamin A (Thermo Fisher Scientific, #121587-10), 1% (v/v) NEAA solution (Sigma, #M7145), 1% (v/v) penicillin–streptomycin (Thermo Fisher Scientific, #15140122). Media was changed daily. On day 30, optic-cup structures were detached from the 6-cm plate by checkerboard scraping using a 200 µL pipette tip and transferred into a 9-cm Petri dish containing 10 mL 3:1-medium. The medium was changed twice per week. Between D36 and D42, retinal structures displaying a bright stratified neuroepithelium were manually picked using an EVOS imaging system (Thermo Fisher Scientific). 3D-retinal organoids were not dissected to remove non-retinal tissue. From day 42 onwards, 3:1-medium was supplemented with 10% (v/v) heat-inactivated FBS (Thermo Fisher Scientific, #10270-106) and 100 µM taurine (Sigma, #T0625- 25G). The medium was changed twice per week. From week 10, 1 µM retinoic acid was added daily (Sigma, #R2625) while the medium was changed twice per week. From week 14, 3D-retinal organoids were cultured in N2-medium consisting of 3 parts DMEM (Thermo Fisher Scientific, #31966047) and one-part F12 medium (Ham’s F-12 Nutrient Mix, Thermo Fisher Scientific, #31765-027) supplemented with 1 × N2 supplement (Gibco, #17502-48), 1% (v/v) NEAA solution (Sigma, #M7145), 1% (v/v) penicillin–streptomycin (Thermo Fisher Scientific, #15140122), 10% (v/v) heat-inactivated FBS (Thermo Fisher Scientific, #10270-106), and 100 µM taurine (Sigma, #T0625-25G). The retinoic acid concentration was reduced to 0.5 µM and added daily. Organoids were cultured at 37 °C and 5% CO_2_ in a humidified incubator (BINDER C150).

#### Microglia precursor cell differentiation

The differentiation protocol is identical to the “Retinal organoid differentiation” section with the following modifications: 2 wells of the hIPSC line tdTomato-SC102A were used to start the differentiation. On day 1, a final concentration of 12.5 ng/mL recombinant human BMP4 (Bone Morphogenetic Protein 4, Peprotech, #120-05) was added to the medium. From day 8 onwards, NIM was changed twice per week. From day 16 until the termination of the differentiation, cultures were maintained in 3:1-medium, with the medium changed twice per week.

#### Harvesting microglia precursor cells (preMG)

From day 36 onwards, preMG were harvested from the supernatant. For this, the supernatant was passed through a 100 µm cell strainer (Corning, ##352360) and collected in a falcon. After centrifugation (VWR, Mega Star 3.0R) at 200*g* for 3 min, the medium was aspirated, and preMG were resuspended in 3:1-medium supplemented with 50 ng/mL recombinant human MCSF (Macrophage Colony Stimulating Factor, BioLegend, #574804). Cells were counted using an automated cell counter (Bio-Rad, #1450102).

#### Neural progenitor cell (NPC) differentiation

According to the manufacturer’s instructions, neuronal progenitor cells (NPCs) were generated using the STEMdiff™ SMADi Neural Induction Kit (STEMCELL Technologies, #08581). NPCs were expanded in STEMdiff™ Neural Progenitor Medium (STEMCELL Technologies, #05833) and frozen in STEMdiff™ Neural Progenitor Freezing Medium (STEMCELL Technologies, #05838). NPCs were passaged at 1.25 × 10^5^ cells/cm^2^ weekly with Accutase™ (STEMCELL Technologies, #07920).

#### Astrocyte differentiation

Astrocytes were differentiated as described previously [142] with minor modifications. For astrocyte differentiation, NPCs were dissociated into single cells using Accutase™ (STEMCELL Technologies, #07920) treatment for 5–10 min. Cells were centrifuged at 400×*g* for 5 min (VWR, Mega Star 3.0R), and the medium was aspirated. Cells were resuspended in complete astrocyte-medium composed of astrocyte medium (Sciencell, #1801-b), 2% (v/v) heat-inactivated FBS (Thermo Fisher Scientific, #10270-106), 1% (v/v) astrocyte growth supplement (Sciencell, #1852) and 1% (v/v) penicillin–streptomycin (Thermo Fisher Scientific, #15140122). 1.5 × 10^5^ cells were seeded per well of Matrigel-coated 6-well plates (Corning, #3516). Cells were cultured for 30 days for astrocyte maturation, and the medium was changed every second day. Cells were passaged before reaching 80–90% confluency once per week. Following the initial 30-day differentiation period, astrocytes were maintained in serum-free astrocytes-medium. Before stimulation, the astrocyte medium was changed gradually to N2-medium over 4 days.

#### Generating microglia-assembled retinal organoids (iMG-_3D_RO)

3D-retinal organoids (_3D_RO) were individually placed into 1.5 mL tubes (Roth, #1KP0.1), each containing 500 µL medium (either supplemented 3:1-medium or N2-medium depending on the age of the differentiation). preMG were collected as described in “Harvesting microglia precursor cells (preMG),” and 6 × 10^4^ cells were added to each organoid. After 72 h, 6–8 organoids were pooled into one well of a 24-well plate (Corning, #3527) and cultured in the respective medium supplemented with 50 ng/mL MCSF for 3 weeks. Media was exchanged twice per week, and retinoic acid was added daily. Cultures were maintained at 37 °C and 5% CO_2_ in a humidified incubator (BINDER C150).

### Generation of dissociated retinal organoid cultures

#### Retinal cup dissociation and 2D plating

At day 105, four 3D-retinal organoids were dissected to remove non-retinal tissue using a scalpel (Fisher Scientific, #11798343), transferred into a 1.5 mL tube, and washed twice in DPBS (Thermo Fisher Scientific, #14190250). Organoids were incubated in Accutase (STEMCELL Technologies, #07920) for 30 min at 37 °C. Then, an equal volume of HBSS (Thermo Fisher Scientific, #14175-129) supplemented with 10% (v/v) heat-inactivated FBS (Thermo Fisher Scientific #10270106) was added, and organoids were dissociated by pipetting up and down ten times using a 200 µL tip. Cells were centrifuged at 3.2×*g* (VWR, Micro Star 17) for 2 min, the medium aspirated, and cells resuspended in N2-medium. Following another centrifugation at 3.2×*g* for 2 min, cells were resuspended in N2-medium supplemented with 20 ng/mL BDNF (Brain-Derived Neurotrophic Factor, Biolegend, #788904), passed through a 70 µm cell strainer (Corning, #352350), and distributed in 6 wells of a Matrigel-coated 8-well chamber (IBIDI, #80826). N2-medium supplemented with 20 ng/mL BDNF was changed every 3–4 days. 0.5 µM retinoic acid was added daily.

#### Integrating microglia precursor cells into dissociated retinal organoids

At day 130, preMG were harvested as described in “Harvesting microglia precursor cells (preMG)” and 6 × 10^4^ cells were added per well of an 8-well chamber. Cultures with and without microglia were maintained in N2-medium supplemented with 20 ng/mL BDNF and 50 ng/mL MCSF. Medium was exchanged every 3–4 days, and 0.5 µM retinoic acid was added daily.

**Table:** 

Plate	Surface area	Retinal cups/well	preMG/well
8 well IBIDI	1 cm^2^	0.75	60,000
24 well	1.9 cm^2^	1.5	125,000
12 well	3.5 cm^2^	3	250,000

### Stimulation paradigms

POLY(I:C) (Tocris, #4287) was diluted in fresh medium with a final concentration of 50 µg/mL and then applied to the cells as indicated in the experiments. For POLY(I:C), POLY(I:C) + IBU, or POLY(I:C) and SC-560, 50 µg/mL POLY(I:C) was mixed with 400 µM of the active enantiomere S(+)-ibuprofen (Sigma-Aldrich, #375160-1G) [143] or with 20 nM SC-560 (Abcam, # ab120649) in fresh medium and applied to the cells.

#### For microglia precursor cells to assay gene expression

preMG were harvested as described “Harvesting microglia precursor cells (preMG)” and 2 × 10^5^ cells were transferred into one well of a 24-well (Corning, #3527) containing 3:1-medium supplemented with 50 ng/mL MCSF. After 24 h, the medium was replaced, and preMG were treated as described above. Untreated controls received 3:1-medium supplemented with 50 ng/mL MCSF. preMG were incubated for 24 h at 37 °C and 5% CO_2_. Four distinct cultures of independent differentiations were analyzed per condition.

#### For differentiated astrocytes

80% confluent astrocyte cultures were stimulated as described above. Three distinct cultures of independent differentiations were analyzed per condition.

#### *For assembled dissociated retinal organoids (*_*diss*_*RO) with and without microglia*

At week 20 (D139), N2-medium supplemented with 50 ng/mL MCSF was changed. We omitted the BDNF application because it has been shown to have anti-inflammatory effects [17, 18]. The withdrawal did not significantly alter ganglion cell survival (data not shown).

For each condition, one well of _diss_RO and iMG-_diss_RO were treated for 24 h as described above. Untreated controls received medium supplemented with 50 ng/mL MCSF. Five distinct cultures of independent differentiations were analyzed per condition.

#### For retinal organoids

At week 20 (D139), three 3D-retinal organoids were transferred into one well of a 24-well plate per condition and cultured in N2-medium supplemented with 50 ng/mL MCSF. _3D_ROs in parallel to iMG-_3D_ROs were treated as described above. Untreated controls received N2-medium supplemented with 50 ng/mL MCSF. Organoids were incubated for 24 h at 37 °C and 5% CO_2_. Five distinct cultures of independent differentiations were analyzed per condition.

#### For CCL2 stimulation

At week 20 (D139), N2-medium supplemented with 50 ng/mL MCSF was changed, and BDNF was withdrawn from the medium. For each condition, one well of _diss_RO and iMG-_diss_RO were treated with recombinant human CCL2 (C–C Motif Chemokine Ligand 2, Peprotech, #300-04) at a final concentration of 10 ng/mL, 20 ng/mL, or 50 ng/mL. Untreated controls received N2-medium supplemented with 50 ng/mL MCSF. Five distinct cultures of independent differentiations were analyzed per condition.

### Gene expression profile of microglia marker and inflammatory cytokines

#### Cultures to determine iMG marker

Cultures were prepared as described in the sections “Retinal cup dissociation and 2D plating” and “Integrating microglia precursor cells into dissociated retinal organoids” with the following modification: Samples were grown in 24-well plates. The number of integrated iMG was constant between 1 and 10 days of co-culture (data not shown).

#### RNA isolation

Samples were washed with DPBS (Thermo Fisher Scientific, #14190250) before RNA isolation using the innuPREP RNA Mini Kit 2.0 (Analytik-Jena, #845-KS-2040050) as described in the manufacturer's instructions. cDNA synthesis was performed with LunaScript RT SuperMix Kit (New England Biolabs, #E3010L) with a total RNA amount of 200–800 ng (same amount for each condition within experimental repetition) and stored at − 20 °C.

#### Gene expression analysis

RT-qPCR (Luna Universal qPCR Master Mix, New England BioLabs, #M3003L) was performed in 384-well plates (Bio-Rad; HSR4805) using the Roche Lightcycler 480 applying the device’s “Second Derivative Maximum Method.” The total reaction volume was 10 µL containing 1 µL of 1:10 diluted cDNA. The final concentration for each primer was 0.25 µM. The primer pairs are listed in Supplementary Table 2. Cycle conditions were 60 s at 95 °C for initial denaturation, followed by 40 cycles of denaturation (15 s; 95 °C) and annealing/extension (30 s; 60 °C). Each run was completed with a melting curve analysis to confirm the amplification of only one amplicon. Each PCR reaction was run in triplicates, from which a mean Cq value was calculated and used for further analysis. dCq values were obtained by normalizing mean Cq values to the geometric mean of four reference genes (GAPDH, ACTB, OAZ1, RPL27) measured within the same sample (Eq. 1). ddCq values were then calculated by normalizing dCq values to the respective control condition (untreated cells/organoids) within each experimental repetition (Eq. 2). Fold changes were obtained by transforming ddCq values from log2 to linear scale (Eq. 3).

Equations for consecutive RT-qPCR normalization:

1 $$_{{\text{d}}} {\text{Cq}} = {\text{Geometric}}\;{\text{mean}}_{{\text{reference genes}}} - {\text{Cq}}$$

2 $$_{{{\text{dd}}}} {\text{Cq}} =_{{\text{d}}} {\text{Cq}} -_{{\text{d}}} {\text{Cq}}_{{{\text{control}}}}$$

3 $${\text{Fold}}\;{\text{change}} = {2}^{{{\text{ddCq}}}}$$

To analyze the mRNA expression relative to GAPDH, dCq values were normalized to GAPDH dCq values in the respective conditions.

### Proteome profiler array

Cultures were prepared as described in the sections “Retinal cup dissociation and 2D plating” and “Integrating microglia precursor cells into dissociated retinal organoids” with the following modification: Since samples were grown in 12-well plates, three retinal cups were dissociated per well, and 2.5 × 10^5^ preMG were added per well. Cultures were stimulated as described in “Stimulating microglia assembled dissociated retinal organoids”.

#### Human cytokine array

After two, four, or 24 h of stimulation, the supernatant was harvested, snap-frozen on dry ice, and stored at − 80 °C. The Proteome Profiler Human Cytokine Array Kit (R&D Systems, #ARY005B) was performed following the manufacturer’s instructions. The membranes were imaged using the luminescent image analyzer Amersham Imager 600 (GE Healthcare Bio-Science). For the 24-h time point, the supernatant from three distinct cultures of independent differentiations was assayed. Only one supernatant from one differentiation was screened for the 2 and 4-h time points.

#### Analysis of proteome profiler array

Pixel densities for positive signals were extracted using the ImageJ plugin ‘Protein Array Analyzer’ (https://imagej.nih.gov/ij/macros/toolsets/Protein%20Array%20Analyzer.txt). For each experimental condition, the average signals were determined per protein-of-interest, background signal subtracted, and signals normalized to the mean of six reference spots per membrane. Hierarchical clustering of the median (24 h time point) of normalized pixel values was carried out using the *pheatmap* package (version 1.0.12, RRID: SCR_016418) in R (version version 4.2.2). Fold changes were obtained by normalizing relative pixel values to the control condition.

### ELISA

Cultures were stimulated as described in “Stimulating microglia assembled dissociated retinal organoids” or “Stimulating retinal organoids” After 24 h of stimulation, the supernatant was harvested, snap-frozen on dry ice, and stored at − 80 °C. PGE2 ELISA (Enzo Life Sciences, #ADI-900-001) was performed according to the manufacturer’s instructions. Samples were analyzed in duplicates, and PGE2 concentration was determined based on the standard curve. The supernatant from three distinct cultures of independent differentiations was assayed.

### Histology

#### Fixation of 3D-retinal organoids

3D-retinal organoids were fixed in 4% (w/v) PFA (Paraformaldehyde, Thermo Fisher Scientific, #28908) in PBS for 25 min at room temperature on an orbital shaker in the dark. Then, organoids were washed three times with PBS at room temperature and cryopreserved in 30% (w/v) sucrose (Sigma-Aldrich, #84097) in PBS overnight at 4ºC or stored in PBS at 4 °C until further use.

#### Fixation of microglia precursor cells and dissociated retinal organoids

Cells were fixed in 4% (w/v) PFA in PBS for 20 min at room temperature in the dark, then washed three times with PBS at room temperature and stored in PBS at 4 °C.

#### Cryostat sectioning

Cryopreserved 3D-retinal organoids were transferred to a cryomold (PolyScience, #18985) using a 1250 µL wide orifice pipette tip and embedded in Tissue-Tek O.C.T. compound (TTEK, A. Hartenstein) on dry ice. Samples were stored at − 80 °C until further use. Organoids were cut into 50 µm sections using a cryostat (MICROM, NX70 CRYOSTAR, Thermo Scientific). Sections were mounted onto glass slides Superfrost Plus (Lactan, #H867.1), dried at room temperature overnight, and stored at − 80 °C until further use. For immunostainings, slides were thawed and dried for 1 h at room temperature. Sections on glass slices were encircled with an engraving, hydrophobic pen (Sigma-Aldrich, #Z225568).

#### Immunostaining of cryostat sections, microglia precursor cells, and dissociated retinal organoid cultures

Samples were incubated in a “blocking solution” containing 1% (w/v) bovine serum albumin (Sigma, #A9418), 5% (v/v) Triton X-100 (Sigma, #T8787), 0.5% (w/v) sodium azide (VWR, #786-299), and 10% (v/v) serum (either goat, Millipore, #S26, or donkey, Millipore, #S30) for 2 h in a humidified chamber protected from light at room temperature. Afterward, the samples were immunostained with primary antibodies diluted in antibody solution containing 1% (w/v) bovine serum albumin, 5% (v/v) triton X-100, 0.5% (v/v) sodium azide, 3% (v/v) goat or donkey serum. They incubated overnight in a humidified chamber at room temperature. For the list of primary antibodies, see Supplementary Table 3. After washing the samples three times with PBS, the samples were incubated light-protected in a humidified chamber for 2 h at room temperature, with the secondary antibodies diluted in antibody solution. The secondary antibodies raised in goat or donkey were purchased from Thermo Fisher Scientific (Alexa Fluor 488, Alexa Fluor 568, Alexa Fluor 647, 1:2000). The sections were washed three times with PBS. The nuclei were labeled with Hoechst 33342 (Thermo Fisher Scientific, Cat#H3570, 1:5000 diluted in PBS) for 15 min and then washed two times in PBS. After immunostaining, antifade solution [10% (v/v) mowiol (Sigma, #81381), 26% (v/v) glycerol (Sigma, #G7757), 0.2 M tris buffer pH 8, 2.5% (w/v) Dabco (Sigma, #D27802)] was dropped on the cryostat sections and covered with microscope coverslips (Menzel-Glaser #0). Slides were dried at room temperature overnight. 8-well chambers were maintained in PBS. Samples were kept at 4 °C for long-term storage.

#### Immunostaining of entire 3D-retinal organoids

The staining was performed as described under “*Immunostaining of cryostat sections, microglia precursor cells and dissociated retinal organoid cultures*” with the following adaptations: 3D-organoids were incubated in blocking for 24 h on an orbital shaker at 4 °C in the dark. The primary antibody concentration was doubled, and organoids were incubated for 10 days on an orbital shaker at 4 °C in the dark. After washing the organoids three times in PBS for 30 min each, secondary antibodies (Thermo Fisher Scientific, Alexa Fluor 488, and Alexa Fluor 647, 1:500) and Hoechst 33342 (1:1000) diluted in antibody solution were added for 3 days on an orbital shaker at 4ºC in the dark. Finally, the organoids were washed three times in PBS for 30 min each. 4–5 3D-organoids were placed into one well of an 8-well chamber (IBIDI, #80826) and covered with 3% (w/v) low gelling temperature agarose (Sigma-Aldrich, #A9414-25G). The samples were stored in glycerol (Sigma-Aldrich, G7757) overnight at 4 °C in the dark.

### Imaging and image analysis

#### Brightfield

The differentiation was monitored using a bright-field microscope (Olympus CKX41) with 5×, 10× and 20× objectives (Olympus) and a lens marker (Nikon), and an EVOS imaging system (Thermo Fisher Scientific) with 2×, 4×, 10×, 20×, 40× objectives (Thermo Fisher Scientific).

#### Confocal microscopy

Images were acquired with a Zeiss LSM880 Airyscan or LSM800 inverted. For overview images, Plan-Apochromat 10× air objective NA 0.45 (WD = 2.1 mm) or Plan-Apochromat 20× Air objective NA 0.8 were used, and z-stacks were acquired. For detailed images, Plan-Apochromat 40× oil immersion objective NA 1.3 was used. For synaptic puncta analysis, images were acquired on Zeiss LSM900 microscope using a Plan-Apochromat 40X objective NA 1.4 using ‘confocal’ mode.

#### Imaging of dissociated retinal organoids

Three regions-of-interests were acquired per condition and biological replicate using the Plan-Apochromat 20× Air objective NA 0.8 with a zoom of 0.8.

#### Imaging of 3D retinal organoid sections

Based on the nuclei staining, one cryostat section per 3D retinal organoid displaying a retinal cup with a lumen was identified and imaged using the Plan-Apochromat 20× Air objective per condition and biological replicate.

#### Imaging of entire 3D-retinal organoids

The embedded organoids were imaged using Plan-Apochromat 10× Air objective NA 0.45 (WD = 2.1 mm).

#### Live cell imaging of dissociated microglia assembled retinal organoids

Microglia-assembled dissociated retinal organoids were generated as described in “Incorporating microglia-like cells into dissociated retinal organoids”. The cells were stimulated as described in “Stimulating microglia assembled dissociated retinal organoids”. Images were acquired with a Zeiss LSM880 inverted microscope and a Plan-Apochromat 20×/NA 0.8 Air objective in a temperature-controlled chamber (37 °C). Z-stacked images of the Alexa 568 channel were captured every minute for 20 min.

#### Image analysis

Confocal images were converted to.ims files using the Imaris File Converter 9.9.1 and imported to Imaris 9.9 (Bitplane Imaris 3/4D Image Visualization and Analysis Software). Images were cropped and processed using background subtraction.

#### iMG positioning within layers

Cryostat sections with a focus on retinal cups were used for the analysis. Since we focused on retinal cups displaying a laminated structure, Hoechst-staining was used to identify the formation of layers. Microglia positioning was based on the location of the microglial cell soma. Each data point represents the percentage of microglia within a respective layer relative to the total number per section.

#### Determining the number of microglia in entire 3D-retinal organoids

Z-stack images of entire organoids were cropped to focus on the retinal cup. The number of microglia-like cells (iMG) were determined using the spot function of Imaris. The estimated XY diameter was set to 15 µm.

#### Determining cell numbers

The spot function of Imaris was used to analyze cells of interest and Hoechst^+^-cells. For nuclear stainings such as Hoechst, OTX2, BRN3, and KI67, the estimated XY diameter was set to 7 µm. For CALB2, CALB1, and RCVRN-labeling, the estimated XY diameter was set to 10 µm. To analyze the number of tdTomato^+^-iMG, the estimated XY diameter was set to 15 µm. The spots were manually edited. For cryostat sections of retinal organoids, we focused on the retinal cup.

For analysis, each data point represents the percentage of the respective marker relative to the total number of Hoechst^+^-cells, or the total number of microglia was determined for each region of interest. To calculate the proliferation rate of retinal cell types, the number of KI67^+^/iMG was subtracted from the total number of KI67^+^-cells, and the number of iMG were subtracted from the total number of Hoechst^+^-cells.

Fold change was determined by normalizing the median of three regions of interest per biological replicate to the respective control condition (untreated cells).

#### Determining Hoechst^+^-fragments

Hoechst^+^-fragments with a 2–5 µm diameter were manually counted using Imaris software. In the plot, each data point represents the number of Hoechst^+^-fragments per mm^2^.

#### Microglia surveillance

Time-lapse videos were binarized in ImageJ using the method ‘Li.’ The Matlab script determined the surveillance index [144], normalized to the total number of microglia imaged per video. Fold changes were calculated by normalizing to untreated control within each experimental repetition.

#### Microglia morphology

To determine the area, we generated the microglia surface with the surface rendering module with the surface detail set to 0.2 µm in Imaris. Incomplete iMG morphologies at the image border were manually removed and not included in the analysis. We excluded surfaces if multiple microglia were summarized as one surface. The exported Imaris file shows the surface area for each detected surface. To extract the number of end points and the total branch length, images were preprocessed using a Python script. The iMG channel was extracted and the image converted to an 8-bit format. After equalizing the images, a ‘Top-hat filter’ (disk size = 15) and ‘unsharp mask’ (sigma = 3) were applied. The mean threshold function was used to generate binary images. Individual cells were identified, and the morphometric features were calculated from the skeletonized representation using a Python script as described in [145].

#### *Microglia engulfing BRN3*^+^*-cells*

Surface rendering was performed for iMG and BRN3^+^-cells with the surface detail set to 0.2 µm. The surface-surface co-localization function in Imaris was used to visualize co-localization. The total number of iMG and the number of iMG engulfing BRN3^+^-cells were determined using the spot function. In the plot, each data point represents the percentage of iMG engulfing a BRN3^+^-cell relative to the total number of microglia per field of view.

#### Distance from spot to surface

First, all BRN3^+^-cells were determined using the spot function of Imaris (XY diameter = 7 µm). Second, the iMG surface was generated using the surface rendering module with the surface detail set to 0.2 µm. Finally, the function ‘spot to surface’ with a distance of 5 µm was used to determine the number of BRN3^+^-cells close to the iMG surface. In the plot, each data point represents the percentage of BRN3^+^-cells close to iMG relative to the total number of BRN3^+^-cells per field of view.

#### Synaptic puncta quantification

Images were processed using background subtraction and median filter. MAP2 (405/420 nm), VGLUT1 (488/520 nm), and PSD95 (633/650 nm) channels were deconvolved in Imaris using the default parameter settings for oil objectives. For the analysis, we focused on MAP2^+^-cells, cropped one branch (25-35 µm) close to the soma, and measured its length. Then, the MAP2 surface was generated using the surface rendering module with the surface detail set to 0.2 µm. With the Spot function in Imaris, we detected all VGLUT1^+^ (XY diameter = 0.19 µm) and all PSD95^+^ puncta (XY diameter = 0.18 µm) in the field of view. Using the function ‘spot to surface’ with a distance of 0.3 µm, we determined VGLUT^+^-spots or PSD95^+^-spots close to the rendered MAP2-surface and extracted the statistics. Finally, we analyzed the number of active synapses. Therefore, we focused on the puncta close to the MAP2 surface. Using the function ‘colocalization’ with a distance of 0.3 µm, we determined VGLUT^+^-puncta in close proximity to PSD95^+^-puncta. We analyzed three cells per field of view. In the plot, each data point represents the number of VGLUT1^+^/PSD95 ^+^-puncta per 10 µm MAP2^+^-process.

#### Graphics

All graphics were generated using R (version 4.2.2). Excel files were loaded into R via the *xlsx* package (version 0.6.5) [146]. Plots were made using ggplot2 (version 3.4.1) [147].

### Calcium imaging of dissociated cultures

#### AAV production and titration

The ISTA Molecular Biology Facility generated the virus. Human embryonic kidney (HEK) 293T cells were maintained at 37 °C in 5% (v/v) CO_2_ in complete medium (DMEM medium (Thermo Fisher Scientific, #31966047), 10% (v/v) fetal bovine serum (Thermo Fisher Scientific, #10270106], 1% (v/v) penicillin/streptomycin (Thermo Fisher Scientific, #15140122), 1% (v/v) non-essential amino acids (Sigma-Aldrich, #M7145-100ML). Ten 15-cm culture dishes with 80% confluency were transfected using 6.8 μM polyethyleneimine (Polysciences, #24765-1), 70 µg AAV transgene plasmid (pAAV-EF1a-GCaMP6s-WPRE-pGHpA, Addgene, #67526), 70 µg 7M8 Cap-encoding plasmid (7M8, Addgene, #64839), 200 µg pHGT1-Adeno1 helper plasmid. Sixty hours post-transfection, cells were harvested with a cell scraper and pelleted at 4000 rpm for 15 min at 4 °C. The pellet was resuspended in lysis buffer (200 mM NaCl, PBS, 0.001% pluronic F68, sterile filtered). Cell-lysis was obtained by three rounds of freezing–thawing cycles between dry ice/ethanol and a 37 °C water bath, followed by sonication for 1 min at 37 kHz. Next, Benzonase (50 U/mL, Sigma Aldrich, #E1014-25KU) was added, and the solution was incubated at 37 °C for 45 min. Afterward, the solution was centrifuged at 2415×*g* for 10 min at 4 °C. The AAV particles in the supernatant were purified by discontinuous iodixanol gradient ultracentrifugation. Optiseal tubes (Beckman Coulter, 361625) were filled with a density gradient of 60%, 40%, 25%, and 17% iodixanol solutions (Optiprep Iodixanol, Progen Biotechnik, 1114542). The virus lysate was loaded on the top layer, and the tubes were centrifuged at 350,000*g* (Beckman Optima XPN-100 ultracentrifuge Sorvall T-850 rotor) for 90 min at 10 °C. The AAV particles were harvested from the intersection of 60% and 40% gradients and concentrated using 100 kDa Vivaspin 20 Centrifugal Concentrator. Aliquots were stored at − 80 °C.

For titration by qPCR, AAV particles were denatured with DNase I (Fisher Scientific, #10103533) and the viral DNA quantification was performed with Universal SYBR Master Mix 2X (Thermo Fisher Scientific, #4309155) using the following primers: forward primer: 5ʹ-GGAACCCCTAGTGATGGAGTT; reverse primer: 5ʹ-CGGCCTCAGTGAGCGA. The final titer measured 1.1 × 10^13^ viral genome copy number per milliliter (GC/mL).

#### AAV infection of dissociated cultures

At week 17 (D120), dissociated retinal organoid cultures were infected with 5 × 10^10^ GC of AAV2/7m8- EF1α- GCAMP6s [10, 112] cultured in 100 µL 3:1-medium. After 24 h, 100 µL fresh 3:1-medium was added. The next day, the medium was changed to N2-medium supplemented with 20 ng/mL BDNF. The medium was changed every 3 to 4 days, and 0.5 µM retinoic acid was added daily.

#### Calcium imaging

Four days before calcium imaging at day 138, dissociated retinal organoids were gradually transferred to BrainPhys medium (StemCell Technologies, #05791) supplemented with 1 × N2 supplement, 100 μM taurine supplemented with 20 ng/mL BDNF, 50 ng/mL MCSF and 0.5 µM retinoic acid. Twenty-four hours before imaging, the medium was changed, and BDNF was withdrawn. Cultures were treated as described in “Stimulating microglia assembled dissociated retinal organoids”, except that the samples were cultured in a supplemented BrainPhys medium. Live imaging was performed using the Dragonfly microscope (Andor Dragonfly 505, Oxford Instruments) equipped with a heated chamber at 37 °C and CFI P Apochromat 20× NA 0.95/WD 0.95 mm water objective (Nikon, MRD77200). The Andor iXon Ultra 888Ultra EMCCD camera (13 μm pixel size) was used to acquire the 488 nm channel using a pinhole size of 25 µm. The following parameters were used for acquisition: exposure time of 40 ms, EM gain of 100, Laser 7.0%, and an AOI of 1024 × 1024. Baseline activity was acquired for 5 min using a time series at 12.16 Hz. Baseline calcium dynamics were recorded for 5 min from five distinct cultures of independent differentiations.

#### Pharmacological manipulation

First, baseline calcium activity was recorded for 2.5 min. For pharmacological manipulation, either 1 µM Tetrodotoxin (Abcam, # ab120054) to block voltage-gated sodium channels was applied or a mixture of 10 μM NBQX (2,3-dioxo-6-nitro-7-sulfamoyl-benzo[f]quinoxaline; Tocris Bioscience, #1044), 10 μM DL-AP4 (DL-2-amino-4-phosphonobutyric acid, Tocris Bioscience, #0101), 10 μM (±)-CPP (3-[(R)-2-carboxypiperazin-4-yl]-propyl-1-phosphonic acid; Abcam, #ab144495) to inhibit glutamatergic synaptic transmission was applied. After 5 min of incubation, calcium activity was recorded for another 2.5 min.

To chelate extracellular calcium, 5 mM EGTA (ethylene glycol-bis(β-aminoethyl ether)-*N*,*N*,*N*′,*N*′-tetraacetic acid) was applied and recording immediately continued for another 2 min.

#### Pharmacological manipulation following POLY(I:C) stimulation

For the stimulation of iMG-_diss_RO, see ‘*Stimulating microglia assembled dissociated retinal organoids’.* Otherwise, following 24 h stimulation, calcium activity was recorded for 5 min. Then, a mixture of 10 μM NBQX (2,3-dioxo-6-nitro-7-sulfamoyl-benzo[f]quinoxaline; Tocris Bioscience, #1044), 10 μM DL-AP4 (DL-2-amino-4-phosphonobutyric acid, Tocris Bioscience, #0101), and 10 μM ( ±)-CPP (3-[(R)-2-carboxypiperazin-4-yl]-propyl-1-phosphonic acid; Abcam, #ab144495) was applied to inhibit glutamatergic synaptic transmission. After 5 min of incubation, calcium activity was recorded for another 5 min.

#### Calcium imaging analysis

Cells showing calcium transients were identified manually as regions of interest using ImageJ, and mean grey values were extracted. We focused on cells where the cell soma and the primary branches were clearly visible. Transients in the background were not included in the analysis. Fluorescent signal time series (F/ΔF: change in fluorescence divided by the mean baseline fluorescence) were calculated for each region of interest. Calcium events were detected in Matlab 2017 using the script ‘PeakCaller’ [148] using the following parameters: required rise = 9% absolute; max. lookback = 100 pts; required fall = 5% absolute; max. lookahead = 100 pts; no trend control; trend smoothness = 100; interpolate across closed shutters = true. For each cell, waveform parameters such as number of events and peak amplitude were extracted and plotted.

### Statistical analysis

All statistics were performed using R (version 4.2.2). The linear regression model was calculated using the “lme4”-package. First, groups for comparison were tested for normal distribution and equal variances using the Shapiro–Wilk and Levene tests, respectively. No data was excluded for analysis. If the data was normally distributed and the groups had equal variances, Student’s t-test was used to compare the two groups. For multiple comparisons, the default contrast for unordered variables was set to ‘contr.sum’ to perform one-way ANOVA, followed by Tukey’s post-hoc comparison. Welch’s test was performed to compare normally distributed groups with unequal variances. A two-sided one-sample T-test was used to analyze if a normally distributed condition significantly differs from a value of 1 or 0.

If groups were not normally distributed, the Wilcoxon rank-sum test or Kruskal–Wallis test, followed by Dunn’s test, was used to compare two or multiple groups, respectively. For multiple comparisons, p-values were adjusted using the “p.adjust” function, and the method was set to ‘BH.’ The following packages were used to perform the analysis: “FSA”-package (dunn-test); “multcomp”-package (Tukey’-test); “psych”-package (t-test, kruskal–wallis test); “stats”-package (wilcox-test, shapiro–wilk test), “dplyr”-package (levene-test).

Significance levels are indicated using the following notation: ^n.s.^p > 0.05; ^∗^p < 0.05; ^∗∗^p < 0.01; ^∗∗∗^p < 0.001. Details about the statistical analysis are summarized in Supplementary Table 4, and the respective raw data in Supplementary Table 5.

## Supplementary Information

Supplementary Material 1. **Figure 1. Characterization of 3D retinal organoids.** **a**-**b**, Immunostaining of _3D_RO cryostat sections counterstained with the nuclei-dye Hoechst (blue) and immunostained for **a**, the presynaptic marker VGLUT1 (magenta) and **b**, the postsynaptic marker PSD95 (magenta) at WK13, WK17 and WK20. White arrow: outer plexiform layer forming between the outer- and inner nuclear layer (ONL, INL, respectively). Scale bar: 10 µm. **c**, Representative cryostat section images of 3D-retinal organoid counterstained with the nuclei-dye Hoechst (blue) and immunostained for retinal cell type-specific markers (green) and at week 20: RCVRN (recoverin; photoreceptors). OTX2 (orthodenticle homeobox 2; photoreceptors, bipolar cells). CALB2 (calretinin; photoreceptors, bipolar-, amacrine cells). CALB1 (calbindin; amacrine-, horizontal cells). CHAT (choline acetyltransferase; amacrine cells). BRN3 (brain-specific homeobox/POU domain protein 3B; ganglion cells). RLBP1 (cellular retinaldehyde-binding protein; Müller glia). ONL: outer nuclear layer. INL: inner nuclear layer. White dashed line: outer plexiform layer. #: retinal cup lumen. White arrow: BRN3^+^-cells close to lumen. Scale bar: 50 µm. **d**, Expression of microglia transcript markers in USCS Cell Browser of Cowan et al. [10]: Dataset ID: ‘Developed human retinal organoid.’ Uniform manifold approximation and projection (UMAP) of transcript expression for AIF (also known as IBA1, ionized calcium-binding adapter molecule 1), CX3CR1 (C-X3-C motif chemokine receptor 1), SPI1 (also known as PU.1, Spi-1 proto-oncogene) and P2RY12 (purinergic receptor P2Y12) of 3D-retinal organoid at week 32 and 38. AC: amacrine cell. BC: bipolar cell. Cone: cone photoreceptors. HC: horizontal cell. MC: Müller glia. RPE: retinal pigment epithelium. Rod: rod photoreceptors. Blue dot: not detected. **Figure 2**. Generation of tdTomato^+^-hIPSC cell line and characterization of differentiated tdTomato^+^-microglia precursor cells (preMG). **a** Integration strategy into the adeno-associated virus integration site 1 (AAVS1) locus. DSB: double-strand break. CAG: CMV immediate enhancer/β-actin promoter. HA-L: homologous arm left. HA-R: homologous arm right. HR: homologous recombination. Puro: puromycin selection side. tdTom: tdTomato. **b**, **c**, Validation strategy. Reaction 1: wildtype allele: PCR product 1.4 kbp. Reaction 2: tdTomato allele: PCR product 1.2 kbp. PCR: polymerase chain reaction. **c** PCR product size. Top: Reaction 1-wildtype AAVS1 locus (1.4 kbp). Bottom: Reaction 2 - construct integrated into AAVS1 (1.2 kbp). Orange: wildtype clone. Red: clone with homozygous integration of the construct. NTC: non-template control. Kbp: kilobase pair. **d**, Validating pluripotency for the wildtype human induced pluripotent stem cell (hIPSC) line SC102A (top) and the tdTomato^+^-hiPSC line SC102A (bottom). Immunostaining of hIPSC colonies for NANOG (nanog homeobox, green), OCT3/4 (octamer-binding protein 3, cyan), and counterstaining for the nuclei-dye Hoechst (blue). Intrinsic tdTomato expression (orange). Scale bar: 100 µm. **e**, Bar chart of tdTomato^+^/ IBA1^+^-preMG with standard error of the mean. **f**-**k**, Representative images of tdTomato-expressing microglia precursor cells (preMG, orange) harvested from the supernatant and plated on a new dish. Cells counterstained for the nuclei-dye Hoechst (blue, merged image), immunostained for IBA1 (ionized calcium-binding adapter molecule 1, green) and the microglia/macrophage markers in magenta for **f**, PU.1 (hematopoietic transcription factor PU.1); **g**, RUNX1 (runt-related transcription factor 1); **h**, ITGAM (integrin subunit alpha m); **i**, CD45 (cluster of differentiation 45/ protein tyrosine phosphatase receptor); **j**, CX3CR1 (chemokine (C-X3-C) receptor 1); **k**, MYB (MYB proto-oncogene). Scale bar: 20µm. **Figure 3.** tdTomato^+^-microglia precursor cells (preMG) integration patterns into 3D retinal organoids. Representative images of 3D-retinal organoids. Left: fluorescence image, right: brightfield image. 4x magnification (top) and 10x magnification (bottom). Scale bar: 20µm. **a**, tdTomato^+^-microglia precursor cells (preMG, white arrow) attach on week 17 at the surface of 3D-retinal organoids (_3D_RO). **b**, tdTomato^+^- microglia-like cells (iMG) integrate into the 3D-retinal organoids at week 20, showing a bipolar shape (white arrow). **c**, Images of iMG-_3D_RO cryostat sections with tdTomato^+^-iMG (orange) counterstained with the nuclei-dye Hoechst (blue) and at WK20. White arrowhead: iMG located in the outer plexiform layer forming between the outer- and inner nuclear layers (ONL, INL, respectively). #: lumen. Scale bar: 50 µm. **Figure 4.** Validating iMG signature. **a**, Impact of brain-derived neurotrophic factor (BDNF) on ganglion cell survival. Bar chart of percentage of BRN3^+^-cells relative to Hoechst^+^-cells with standard error of the mean in 3D-retinal organoids (_3D_RO, left) and dissociated retinal organoid culture (_diss_RO, right) cultured either in standard retinal organoid differentiation media without (-BDNF, grey) or supplemented with BDNF (+BDNF, green) from week 15 to 20. _3D_RO: Each dot is one cryostat section of independent retinal cups. Welch's t-test. _diss_RO: Each dot is one region of interest. One-sample Wilcoxon signed rank test. **b**, Image of iMG distribution within iMG-_diss_RO (orange) at WK20, counterstained with the nuclei-dye Hoechst (blue). Scale bar: 100 µm. **c**, Schematic timeline of microglia marker expression during development. **d**, RT-qPCR for the microglia marker C1QA (complement component C1q), CX3CR1 (C-X3-C motif chemokine receptor 1), P2RY12 (purinergic receptor P2Y G-protein-coupled 12) and TMEM119 (transmembrane protein 119) in iMG-_diss_RO after 1 and 10 days of coculture. Bar chart with SEM of mean mRNA transcript expression relative to GAPDH. Each dot is one biological replicate. Student’s t-test. **e-g**, Representative images of iMG-_diss_RO with tdTomato^+^-iMG (orange), counterstained for the nuclei-dye Hoechst (blue) and immunostained in magenta for **e**, early transcription factors PU.1 (hematopoietic transcription factor PU.1), RUNX1 (runt-related transcription factor 1) and MYB (MYB Proto-Oncogene); **f**, ‘early’ microglia marker IBA1 (ionized calcium-binding adapter molecule 1) and CD45 (cluster of differentiation 45/ protein tyrosine phosphatase receptor); and **g**, ‘mature’ microglia marker P2Y12 and TREM2 (Triggering Receptor Expressed On Myeloid Cells 2). Scale bar: 10 µm. For detailed statistical analysis, see Supplementary Table 4. ***p < 0.001. **p < 0.01. *p < 0.05. ^ns^p > 0.05, not significant. **Figure 5.** iMG mediated consequences in the dissociated retinal organoid model. **a-c**, Boxplot of percent of **a**, CCAS3^+^-cells per area; **b**, Hoechst^+^-cells per area and **c**, TX2^+^-photoreceptor- and bipolar cells relative to Hoechst^+^-cells in _diss_RO (grey) and iMG-_diss_RO (orange). **a-b**, Students’s t-test. **c**, Welch's t-test. **d**, Representative images of iMG-_diss_RO for tdTomato expression (orange), counterstained for the nuclei-dye Hoechst (blue) and immunostained for the apoptotic marker CCAS3 (cleaved caspase3, green). White arrowhead: iMG engulfing CCAS3^+^-fragment. Scale bar: 50µm. **e**, Hoechst^+^-fragments (white) in _diss_RO (left) and iMG-_diss_RO (right). Scale bar: 50 µm. **f**, Boxplot of percent of Hoechst^+^-fragments per area in _diss_RO (grey) and iMG-_diss_RO (orange). Welch's t-test. **g**, Representative images of iMG-_3D_RO cryostat sections counterstained with the nuclei-dye Hoechst (blue) and tdTomato^+^-iMG (orange) at WK20. Arrow: iMG engulfing Hoechst^+^-fragment. Scale bar: 50µm. Zoom in: Scale bar: 10µm. Symbols: single ROI of three biological replicates from five independent differentiations. For detailed statistical analysis, see Supplementary Table 4. ***p < 0.001. ^ns^p > 0.05, not significant. **Figure 6. **Individual inflammatory proteome profiler results. **a**, Release of inflammatory cytokines and chemokines into the supernatant based on the experimental paradigm described in Fig. 3a for control (CTRL, grey) and 24h- POLY(I:C) (magenta) stimulation. Individual heatmap plots with color-coded mean pixel intensity relative to the reference of three independent differentiations White: n.d. (not detectable). Side-bar: condition with iMG (orange) *versus* without (white) or CTRL versus POLY(I:C). **b**, Representative membranes for each condition. Numbers refer to the legend below. **c**, Example images of _diss_RO counterstained with the nuclei-dye Hoechst (blue) and immunostained for the glial marker GFAP (glial fibrillary acidic protein, green). Scale bar: 20 µm. **d**, List of proteins assayed on the membrane but not detected in the supernatant of any condition. **Figure 7. **Timeline POLY(I:C)-mediated response. **a**, Same assay as for in Supplementary Figure 6a with additional measurement of cytokine and chemokine release after two and four hours compared to 24h in iMG-_diss_RO with annotated example membranes iMG-_diss_RO. **b**, Boxplot of the total branch length (left) and the number of endpoints (right) per iMG for CTRL (grey) and POLY(I:C) (magenta) following 4h and 24h stimulation in iMG-_diss_RO. iMG were collected from five independent differentiations. Kruskal-Wallis test with post-hoc Dunn’s test. **Figure 8. **The POLY(I:C)-mediated proliferation rate increase cannot be replicated with CCL2 alone. **a**, Experimental timeline. At WK18.5, preMG are added to _diss_RO. After nine days, cultures received fresh medium for control (CTRL, grey) or CCL2 stimulation iMG-_diss_RO for 24 hours. **b**, Effect of CCL2 on retinal cell proliferation excluding iMG. Boxplot of percent KI67^+^-cells relative to Hoechst^+^-cells in iMG-_diss_RO for CTRL and CCL2 stimulation at a final concentration of 10 ng/mL (yellow), 20 ng/mL (orange), and 50 ng/mL (red). Magenta line: Median proliferation rate in POLY(I:C) stimulation of iMG-_diss_RO (Fig. 4g). Symbols: three biological replicates from five independent differentiations. One-way ANOVA. **c**, Effect of CCL2 on iMG proliferation. Boxplot of percent KI67^+^/iMG for CTRL and CCL2 stimulation at a final concentration of 10 ng/mL (yellow), 20 ng/mL (orange), and 50 ng/mL (red). Magenta line: Median iMG-proliferation rate in POLY(I:C) stimulation of iMG-_diss_RO (Fig. 4d). Symbols: three biological replicates from five independent differentiations. Kruskal-Wallis test. For detailed statistical analysis, see Supplementary Table 4. *p < 0.05. ^ns^p > 0.05, not significant. **Figure 9.** Comparison of individual secreted inflammatory mediators after ibuprofen exposure. **a**, Release of inflammatory cytokines and chemokines into the supernatant based on the experimental paradigm described in Fig. 3a for control (CTRL, grey), POLY(I:C) (magenta), and POLY(I:C) and S(+)-ibuprofen (POLY(I:C)+IBU, blue) stimulation. Release of different inflammatory mediators into the supernatant of iMG-_diss_RO. Bar chart of pixel intensity normalized to reference with standard error of the mean for CTRL, POLY(I:C), and POLY(I:C)+IBU. Each symbol: an independent differentiation (n=3). One-way ANOVA with post-hoc Tukey’s test, except IL13, IL18, IL21 Kruskal-Wallis test. For detailed statistical analysis, see Supplementary Table 4. ***p < 0.001. **p < 0.01. *p < 0.05. ^ns^p > 0.05, not significant. **Figure 10. **GCAMP6s expression across retinal cell types. **a**-**e**, Example ROI images of _diss_RO infected with AAV2-GCAMP6s at WK17, analyzed at WK20, counterstained for the nuclei-dye Hoechst (blue) and the calcium sensor GCAMP6s (green), and immunostaining for retinal cell types (magenta): **a**, RCVRN (recoverin; photoreceptors). **b**, OTX2 (orthodenticle homeobox 2; photoreceptors, bipolar cells). **c**, CALB2 (calretinin; photoreceptors, bipolar-, amacrine cells). **d**, CALB1 (calbindin; amacrine-, horizontal cells). **e**, BRN3 (brain-specific homeobox/POU domain protein 3B; ganglion cells). Arrow: Co-expression of calcium sensor and retinal marker. Scale bar: 50µm. **f,** Spontaneous calcium dynamics in iMG-_diss_RO (orange) and _diss_RO (grey) for control (CTRL, grey), POLY(I:C) (magenta), and POLY(I:C) and S(+)-ibuprofen (POLY(I:C)+IBU, blue) stimulation. Boxplot of the mean frequency [Hz] during five minutes of recording. Each dot represents an active cell. Recordings from five biological replicates from independent differentiations. Kruskal-Wallis test. For detailed statistical analysis, see Supplementary Table 4. ^ns^p > 0.05, not significant. **Figure 11. ** PTGS1, PTGS2, and TLR3 mRNA expression profile. **a**-**b**, Expression of (**a**) PTGS1 and PTGS2 (prostaglandin-endoperoxide synthase 1 and 2) as well as (**b**) TLR3 (toll-like receptor 3) in USCS Cell Browser of Cowan et al. [10].

Supplementary Material 2. **Table 1. **Overview of human induced pluripotent stem lines (hIPSC) included in this study.

Supplementary Material 3. Table 2. List of antibodies.

Supplementary Material 4. Table 3. Primer sequences.

Supplementary Material 5.

Supplementary Material 6.

Supplementary Video1_iMG surveillance_control

Supplementary Video2_iMG surveillance_polyIC

Supplementary Video3_iMG surveillance_polyIC_IBU

Supplementary Video4a_Ca dynamics_iMGdissRO_CTRL

Supplementary Video4b_Ca dynamics_dissRO_CTRL

Supplementary Video5_Ca dynamics_TTX

Supplementary Video6_Ca dynamics_glutamatergic

Supplementary Video7_Ca dynamics_EGTA

## Data Availability

Data is provided within the manuscript and the supplementary information files.
