# Supplementary material for: Microglia determine an immune-challenged environment and facilitate ibuprofen action in human retinal organoids
Source: J Neuroinflammation. 2025 Apr 3;22:98. doi: 10.1186/s12974-025-03366-x (PMC11966913; doi:10.1186/s12974-025-03366-x)
Supplement: Supplementary file 1 — Supplementary Material 1. Figure 1. Characterization of 3D retinal organoids. a-b, Immunostaining of 3DRO cryostat sections counterstained with the nuclei-dye Hoechst (blue) and immunostained for a, the presynaptic marker VGLUT1 (magenta) and b, the postsynaptic marker PSD95 (magenta) at WK13, WK17 and WK20. White arrow: outer plexiform layer forming between the outer- and inner nuclear layer (ONL, INL, respectively). Scale bar: 10 µm. c, Representative cryostat section images of 3D-retinal organoid counterstained with the nuclei-dye Hoechst (blue) and immunostained for retinal cell type-specific markers (green) and at week 20: RCVRN (recoverin; photoreceptors). OTX2 (orthodenticle homeobox 2; photoreceptors, bipolar cells). CALB2 (calretinin; photoreceptors, bipolar-, amacrine cells). CALB1 (calbindin; amacrine-, horizontal cells). CHAT (choline acetyltransferase; amacrine cells). BRN3 (brain-specific homeobox/POU domain protein 3B; ganglion cells). RLBP1 (cellular retinaldehyde-binding protein; Müller glia). ONL: outer nuclear layer. INL: inner nuclear layer. White dashed line: outer plexiform layer. #: retinal cup lumen. White arrow: BRN3+-cells close to lumen. Scale bar: 50 µm. d, Expression of microglia transcript markers in USCS Cell Browser of Cowan et al. [10]: Dataset ID: ‘Developed human retinal organoid.’ Uniform manifold approximation and projection (UMAP) of transcript expression for AIF (also known as IBA1, ionized calcium-binding adapter molecule 1), CX3CR1 (C-X3-C motif chemokine receptor 1), SPI1 (also known as PU.1, Spi-1 proto-oncogene) and P2RY12 (purinergic receptor P2Y12) of 3D-retinal organoid at week 32 and 38. AC: amacrine cell. BC: bipolar cell. Cone: cone photoreceptors. HC: horizontal cell. MC: Müller glia. RPE: retinal pigment epithelium. Rod: rod photoreceptors. Blue dot: not detected. Figure 2. Generation of tdTomato+-hIPSC cell line and characterization of differentiated tdTomato+-microglia precursor cells (preMG). a Integration strat [file 12974_2025_3366_MOESM1_ESM.pdf]

Supplementary Figure 1

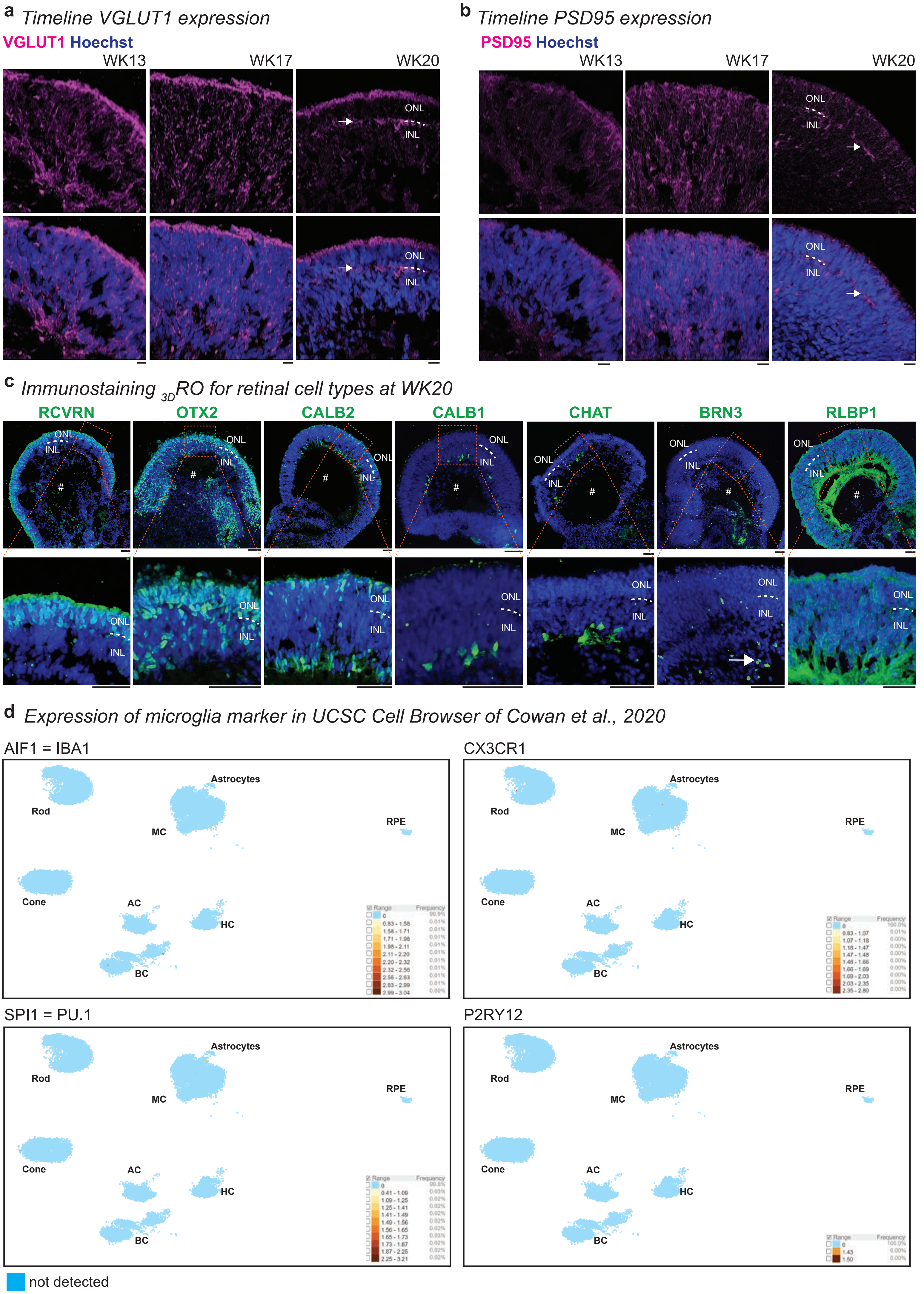

Supplementary Figure 1 – Characterization of 3D retinal organoids.

a-**b**, Immunostaining of 3DRO cryostat sections counterstained with the nuclei-dye Hoechst (blue) and immunostained for **a**, the presynaptic marker VGLUT1 (magenta) and **b**, the postsynaptic marker PSD95 (magenta) at WK13, WK17 and WK20. White arrow: outer plexiform layer forming between the outer- and inner nuclear layer (ONL, INL, respectively). Scale bar: 10 µm.

c, Representative cryostat section images of 3D-retinal organoid counterstained with the nuclei-dye Hoechst (blue) and immunostained for retinal cell type-specific markers (green) and at week 20: RCVRN (recoverin; photoreceptors). OTX2 (orthodenticle homeobox 2; photoreceptors, bipolar cells). CALB2 (calretinin; photoreceptors, bipolar-, amacrine cells). CALB1 (calbindin; amacrine-, horizontal cells). CHAT (choline acetyltransferase; amacrine cells). BRN3 (brain-specific homeobox/POU domain protein 3B; ganglion cells). RLBP1 (cellular retinaldehyde-binding protein; Müller glia). ONL: outer nuclear layer. INL: inner nuclear layer. White dashed line: outer plexiform layer. #: retinal cup lumen. White arrow: BRN3^{+}-cells close to lumen. Scale bar: 50 µm.

d, Expression of microglia transcript markers in USCS Cell Browser of Cowan *et al*., 2020: Dataset ID: 'Developed human retinal organoid.' Uniform manifold approximation and projection (UMAP) of transcript expression for AIF (also known as IBA1, ionized calcium-binding adapter molecule 1), CX3CR1 (C-X3-C motif chemokine receptor 1), SPI1 (also known as PU.1, Spi-1 proto-oncogene) and P2RY12 (purinergic receptor P2Y12) of 3D-retinal organoid at week 32 and 38. AC: amacrine cell. BC: bipolar cell. Cone: cone photoreceptors. HC: horizontal cell. MC: Müller glia. RPE: retinal pigment epithelium. Rod: rod photoreceptors. Blue dot: not detected.

Supplementary Figure 2

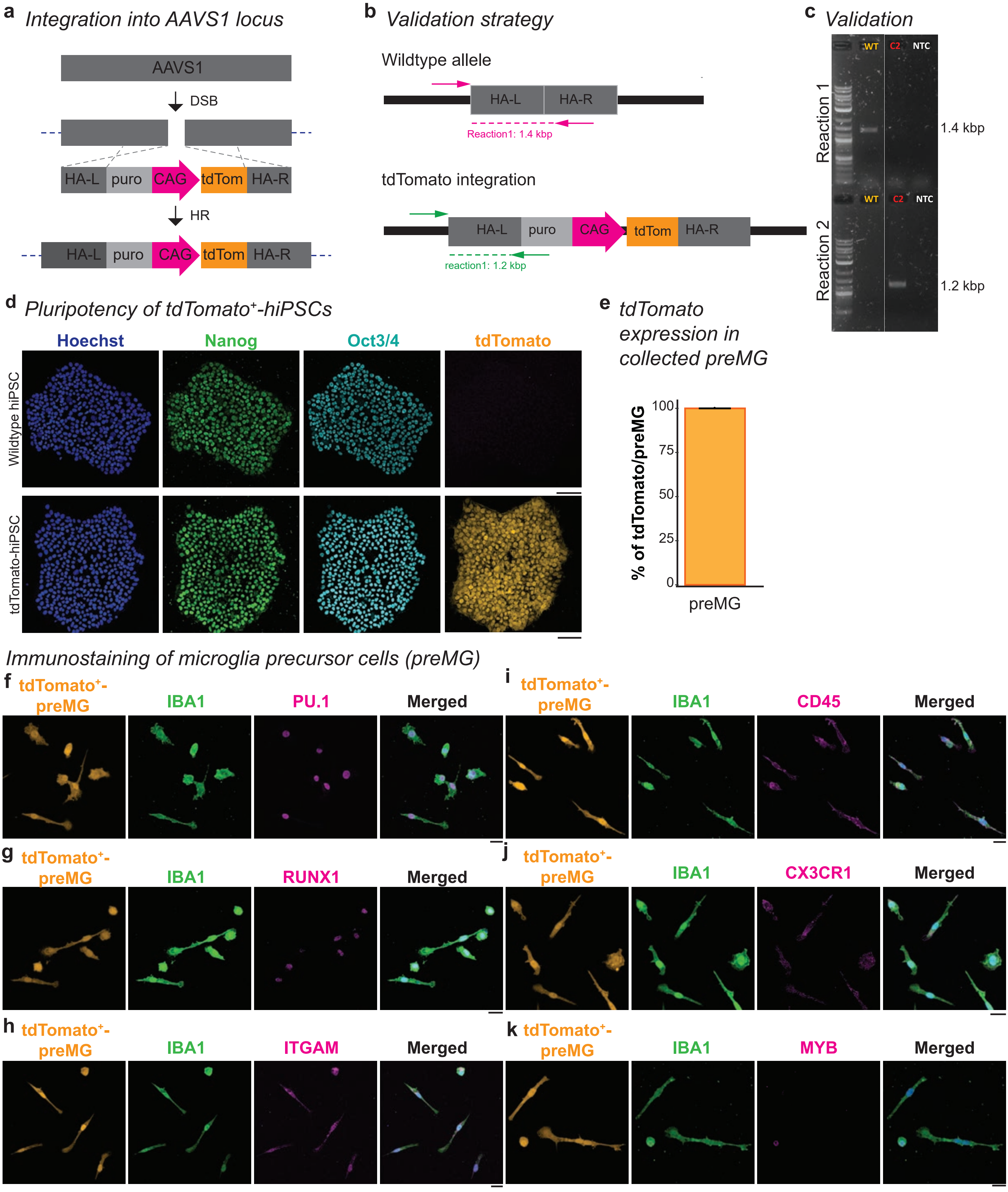

Supplementary Figure 2 – Generation of tdTomato$^+$-hIPSC cell line and characterization of differentiated tdTomato$^+$-microglia precursor cells (preMG).

a, Integration strategy into the adeno-associated virus integration site 1 (AAVS1) locus. DSB: double-strand break. CAG: CMV immediate enhancer/β-actin promoter. HA-L: homologous arm left. HA-R: homologous arm right. HR: homologous recombination. Puro: puromycin selection side. tdTom: tdTomato.

b-**c**, Validation strategy. Reaction 1: wildtype allele: PCR product 1.4 kbp. Reaction 2: tdTomato allele: PCR product 1.2 kbp. PCR: polymerase chain reaction.

c, PCR product size. Top: Reaction 1 - wildtype AAVS1 locus (1.4 kbp). Bottom: Reaction 2 - construct integrated into AAVS1 (1.2 kbp). Orange: wildtype clone. Red: clone with homozygous integration of the construct. NTC: non-template control. Kbp: kilobase pair.

d, Validating pluripotency for the wildtype human induced pluripotent stem cell (hIPSC) line SC102A (top) and the tdTomato$^+$-hiPSC line SC102A (bottom). Immunostaining of hIPSC colonies for NANOG (nanog homeobox, green), OCT3/4 (octamer-binding protein 3, cyan), and counterstaining for the nuclei-dye Hoechst (blue). Intrinsic tdTomato expression (orange). Scale bar: 100 μm.

e, Bar chart of tdTomato$^+$/ IBA1$^+$-preMG with standard error of the mean.

f-**k**, Representative images of tdTomato-expressing microglia precursor cells (preMG, orange) harvested from the supernatant and plated on a new dish. Cells counterstained for the nuclei-dye Hoechst (blue, merged image), immunostained for IBA1 (ionized calcium-binding adapter molecule 1, green) and the microglia/macrophage markers in magenta for **f**, PU.1 (hematopoietic transcription factor PU.1); **g**, RUNX1 (runt-related transcription factor 1); **h**, ITGAM (integrin subunit alpha m); **i**, CD45 (cluster of differentiation 45/ protein tyrosine phosphatase receptor); **j**, CX3CR1 (chemokine (C-X3-C) receptor 1); **k**, MYB (MYB proto-oncogene). Scale bar: 20μm.

Supplementary Figure 3

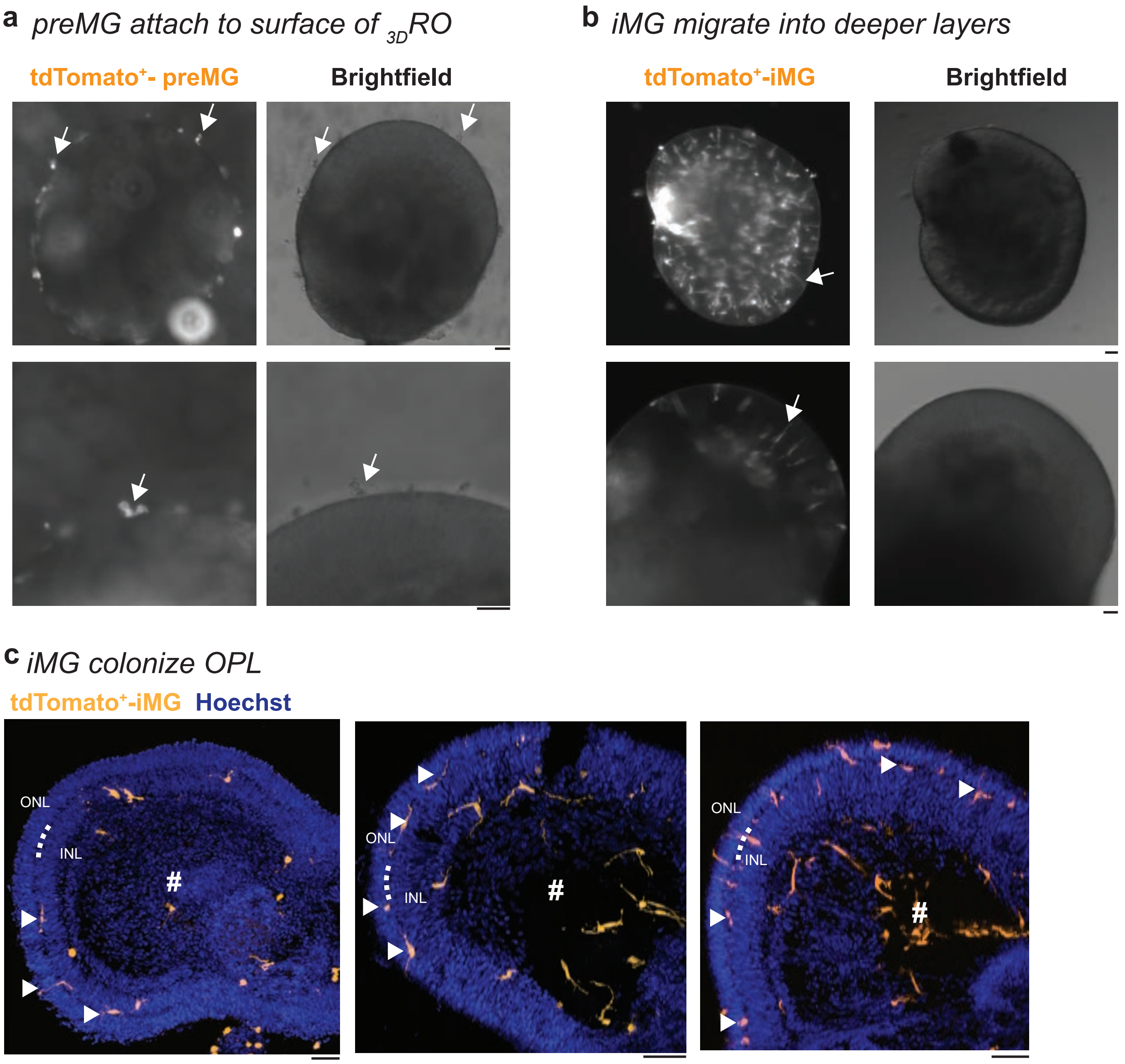

Supplementary Figure 3 – tdTomato^{+}-microglia precursor cells (preMG) integration patterns into 3D retinal organoids.

Representative images of 3D-retinal organoids. Left: fluorescence image, right: brightfield image. 4x magnification (top) and 10x magnification (bottom). Scale bar: 20µm. **a**, tdTomato^{+}-microglia precursor cells (preMG, white arrow) attach on week 17 at the surface of 3D-retinal organoids ($_{3D}$RO). **b**, tdTomato^{+}- microglia-like cells (iMG) integrate into the 3D-retinal organoids at week 20, showing a bipolar shape (white arrow).

c, Images of iMG-$_{3D}$RO cryostat sections with tdTomato^{+}-iMG (orange) counterstained with the nuclei-dye Hoechst (blue) and at WK20. White arrowhead: iMG located in the outer plexiform layer forming between the outer- and inner nuclear layers (ONL, INL, respectively). #: lumen. Scale bar: 50 µm.

Supplementary Figure 4

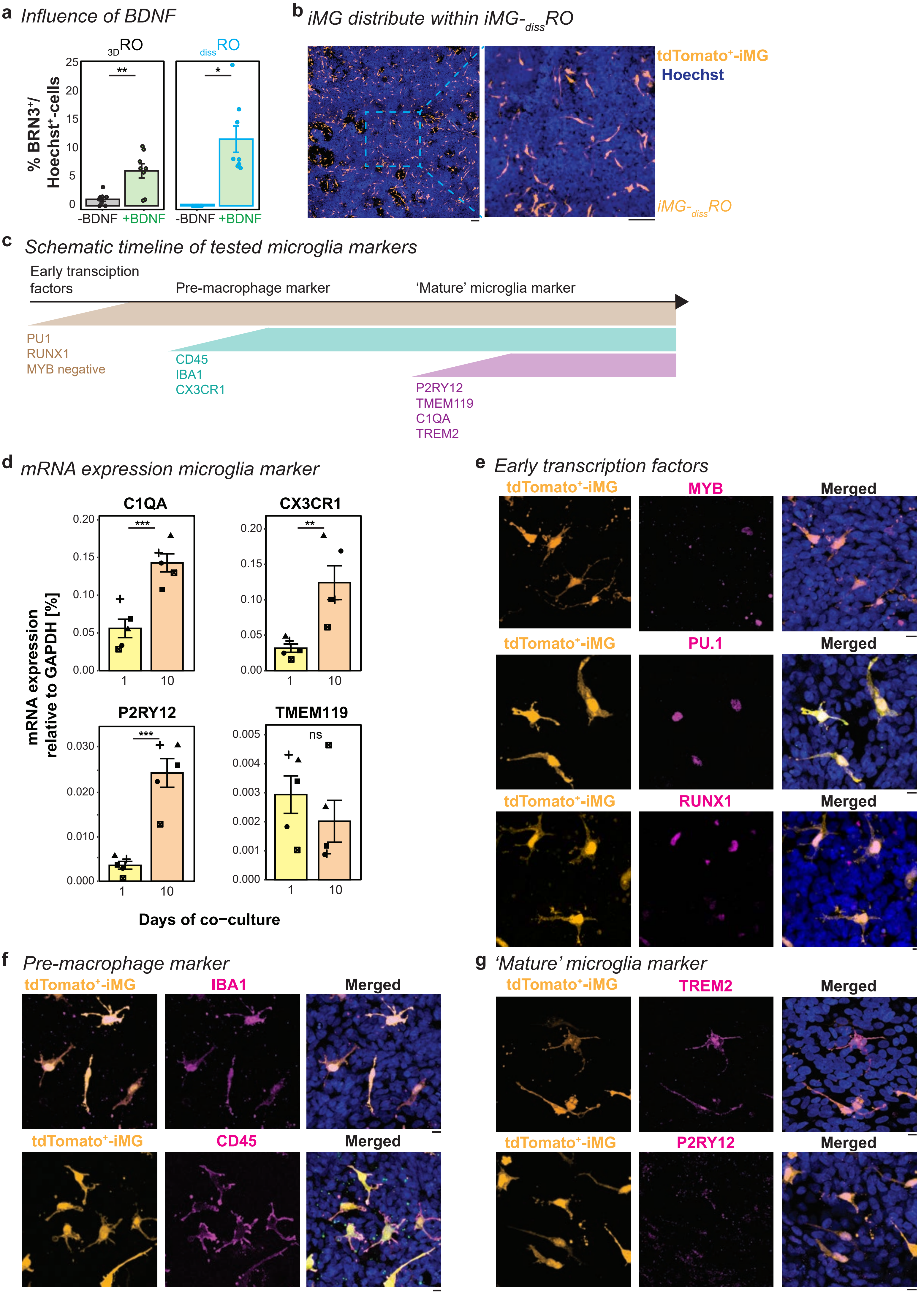

Supplementary Figure 4 – Validating iMG signature.

a, Impact of brain-derived neurotrophic factor (BDNF) on ganglion cell survival. Bar chart of percentage of BRN3^{+}-cells relative to Hoechst^{+}-cells with standard error of the mean in 3D-retinal organoids ($_{3D}$RO, left) and dissociated retinal organoid culture ($_{diss}$RO, right) cultured either in standard retinal organoid differentiation media without (-BDNF, grey) or supplemented with BDNF (+BDNF, green) from week 15 to 20. $_{3D}$RO: Each dot is one cryostat section of independent retinal cups. Welch's t-test. $_{diss}$RO: Each dot is one region of interest. One-sample Wilcoxon signed rank test.

b, Image of iMG distribution within iMG-$_{diss}$RO (orange) at WK20, counterstained with the nuclei-dye Hoechst (blue). Scale bar: 100 µm.

c, Schematic timeline of microglia marker expression during development.

d, RT-qPCR for the microglia marker C1QA (complement component C1q), CX3CR1 (C-X3-C motif chemokine receptor 1), P2RY12 (purinergic receptor P2Y G-protein-coupled 12) and TMEM119 (transmembrane protein 119) in iMG-$_{diss}$RO after 1 and 10 days of coculture. Bar chart with SEM of mean mRNA transcript expression relative to GAPDH. Each dot is one biological replicate. Student's t-test.

e-g, Representative images of iMG-$_{diss}$RO with tdTomato^{+}-iMG (orange), counterstained for the nuclei-dye Hoechst (blue) and immunostained in magenta for **e**, early transcription factors PU.1 (hematopoietic transcription factor PU.1), RUNX1 (runt-related transcription factor 1) and MYB (MYB Proto-Oncogene); **f**, 'early' microglia marker IBA1 (ionized calcium-binding adapter molecule 1) and CD45 (cluster of differentiation 45/ protein tyrosine phosphatase receptor); and **g**, 'mature' microglia marker P2Y12 and TREM2 (Triggering Receptor Expressed On Myeloid Cells 2). Scale bar: 10 µm.

For detailed statistical analysis, see **Supplementary Table 4**.

***$p < 0.001$. **$p < 0.01$. *$p < 0.05$. $^{ns}p > 0.05$, not significant.

Supplementary Figure 5

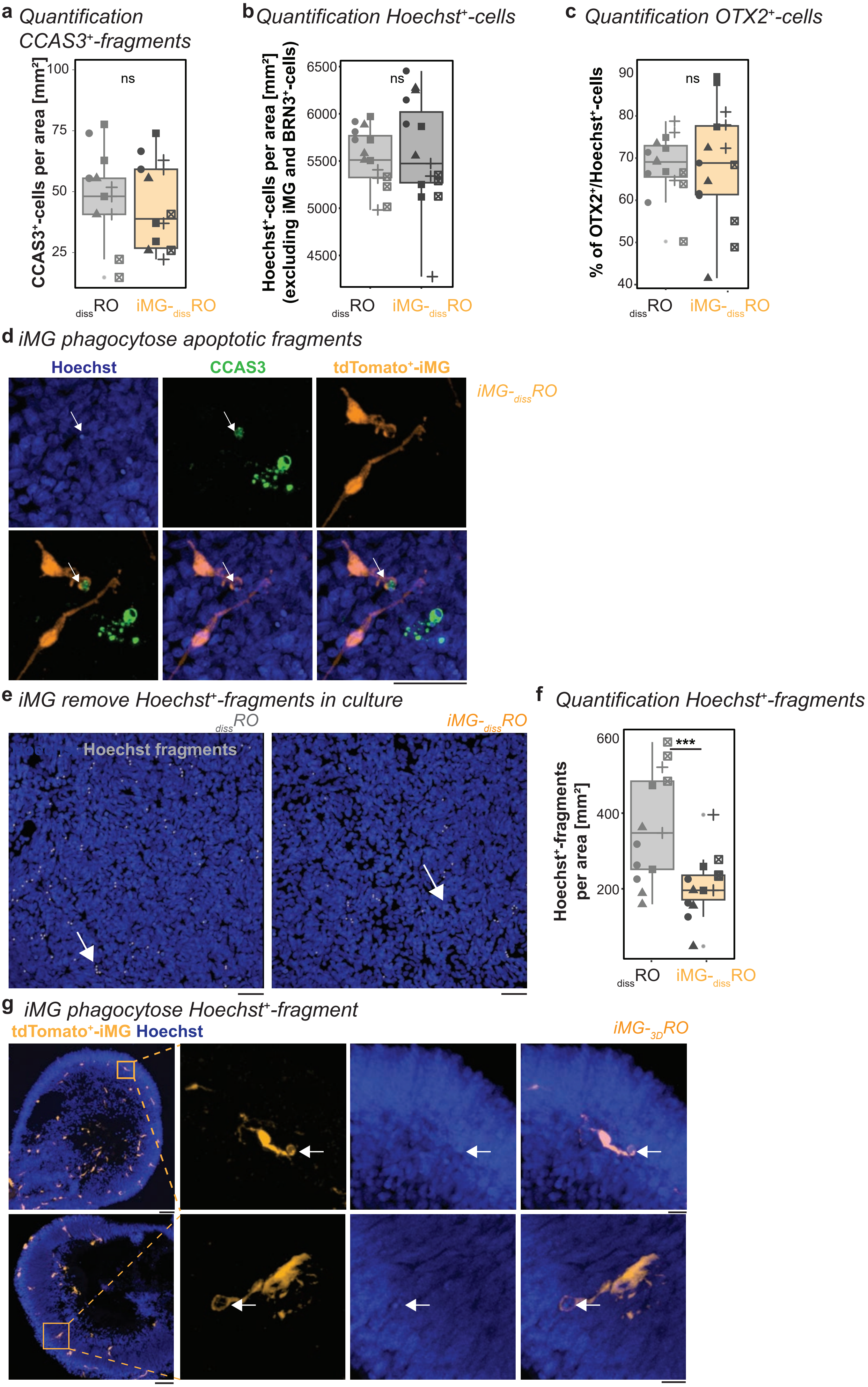

Supplementary Figure 5 – iMG mediated consequences in the dissociated retinal organoid model.

a-c, Boxplot of percent of **a**, $CCAS3^{+}$-cells per area; **b**, $Hoechst^{+}$-cells per area and **c**, $TX2^{+}$-photoreceptor- and bipolar cells relative to $Hoechst^{+}$-cells in $_{diss}RO$ (grey) and iMG-$_{diss}RO$ (orange). **a-b**, Students's t-test. **c**, Welch's t-test.

d, Representative images of iMG-$_{diss}RO$ for tdTomato expression (orange), counterstained for the nuclei-dye Hoechst (blue) and immunostained for the apoptotic marker CCAS3 (cleaved caspase3, green). White arrowhead: iMG engulfing $CCAS3^{+}$-fragment. Scale bar: 50µm.

e, $Hoechst^{+}$-fragments (white) in $_{diss}RO$ (left) and iMG-$_{diss}RO$ (right). Scale bar: 50 µm.

f, Boxplot of percent of $Hoechst^{+}$-fragments per area in $_{diss}RO$ (grey) and iMG-$_{diss}RO$ (orange). Welch's t-test.

g, Representative images of iMG-$_{3D}RO$ cryostat sections counterstained with the nuclei-dye Hoechst (blue) and $tdTomato^{+}$-iMG (orange) at WK20. Arrow: iMG engulfing $Hoechst^{+}$-fragment. Scale bar: 50µm. Zoom in: Scale bar: 10µm.

Symbols: single ROI of three biological replicates from five independent differentiations.

For detailed statistical analysis, see **Supplementary Table 4**.

$^{***}p < 0.001$. $^{ns}p > 0.05$, not significant.

Supplementary Figure 6

a *Individual replicates*

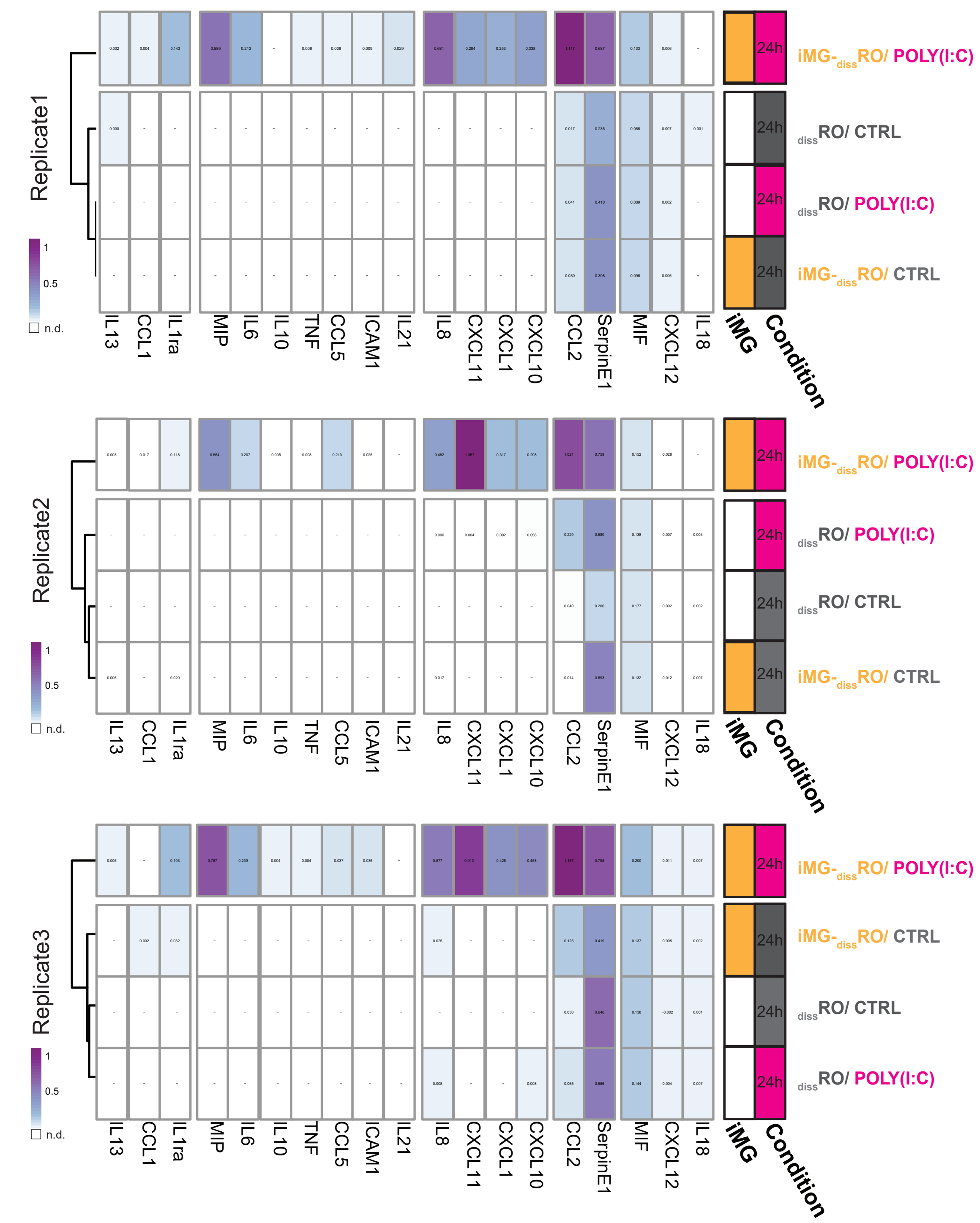

b *Example membranes*

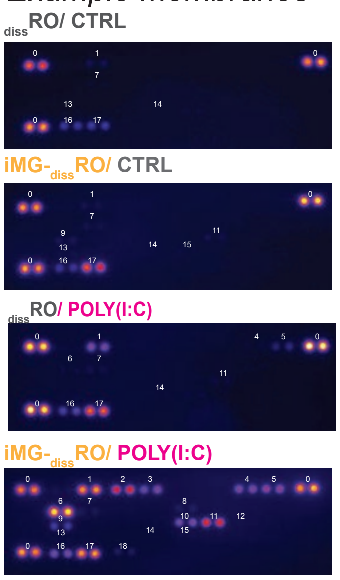

Expressed in at least 1 condition

0 - Reference	10 - IL6
1 - CCL2	11 - IL8
2 - MIP	12 - IL10
3 - CCL5	13 - IL13
4 - CXCL1	14 - IL18
5 - CXCL10	15 - IL21
6 - CXCL11	16 - MIF
7 - CXCL12	17 - SerpinE1
8 - ICAM1	18 - TNF
9 - IL1ra	19 - Negative

c *Glia in $_{diss}$RO*

GFAP Hoechst

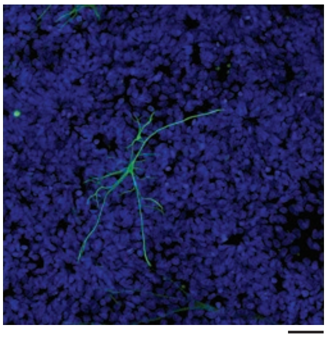

d *Not detected*

CCL1	IL-4
CD40	IL-5
Comp. Comp.	IL-12 p70
C5/C5a	IL-16
G-CSF	IL17A
GM-CSF	IL17E
IFNy	IL-27
IL-1α	IL-32α
IL-1β	TREM1
IL-2	

Supplementary Figure 6 – Individual inflammatory proteome profiler results.

a, Release of inflammatory cytokines and chemokines into the supernatant based on the experimental paradigm described in **Figure 3a** for control (CTRL, grey) and 24h- POLY(I:C) (magenta) stimulation. Individual heatmap plots with color-coded mean pixel intensity relative to the reference of three independent differentiations White: n.d. (not detectable). Side-bar: condition with iMG (orange) *versus* without (white) or CTRL *versus* POLY(I:C).

b, Representative membranes for each condition. Numbers refer to the legend below.

c, Example images of $_{diss}$RO counterstained with the nuclei-dye Hoechst (blue) and immunostained for the glial marker GFAP (glial fibrillary acidic protein, green). Scale bar: 20 μm.

d, List of proteins assayed on the membrane but not detected in the supernatant of any condition.

Supplementary Figure 7

a *Timeline of cytokine release iMG-$_{diss}$RO*

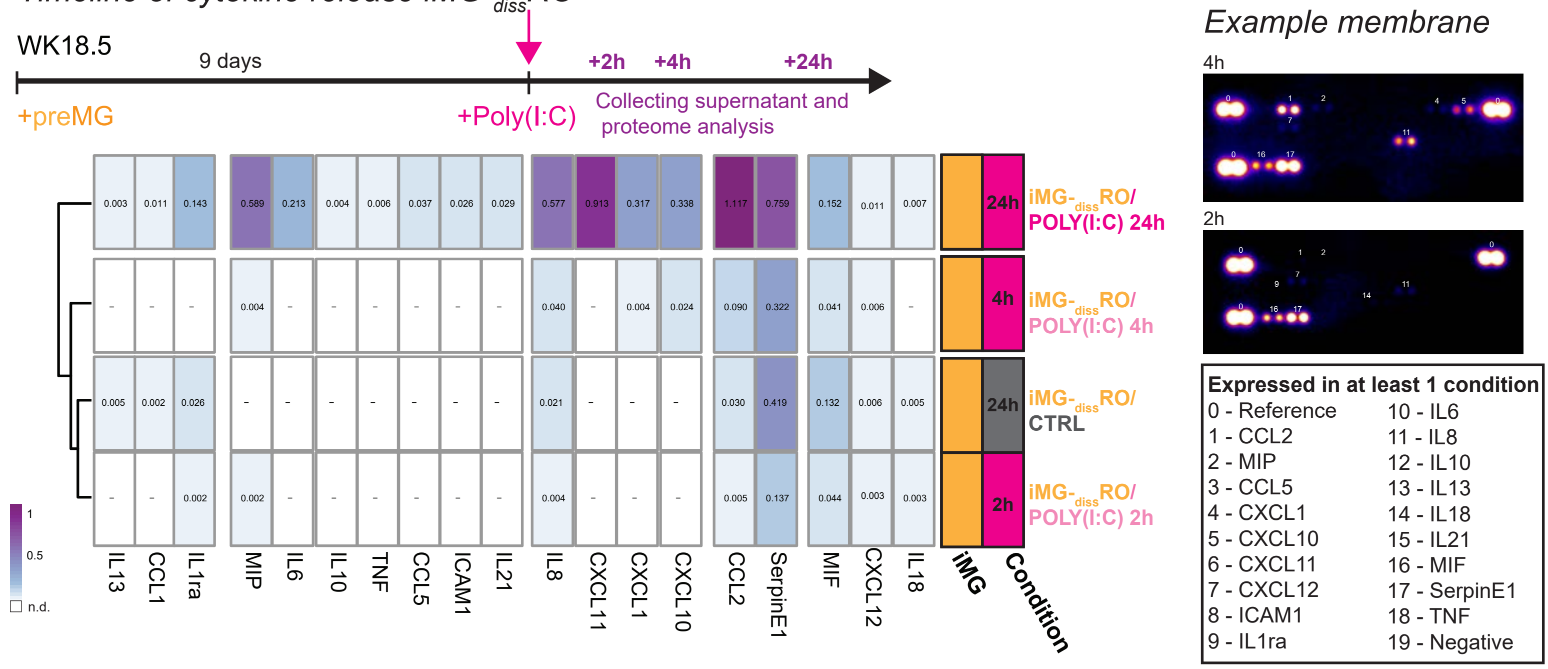

b *Timeline iMG morphology in iMG-$_{diss}$RO*

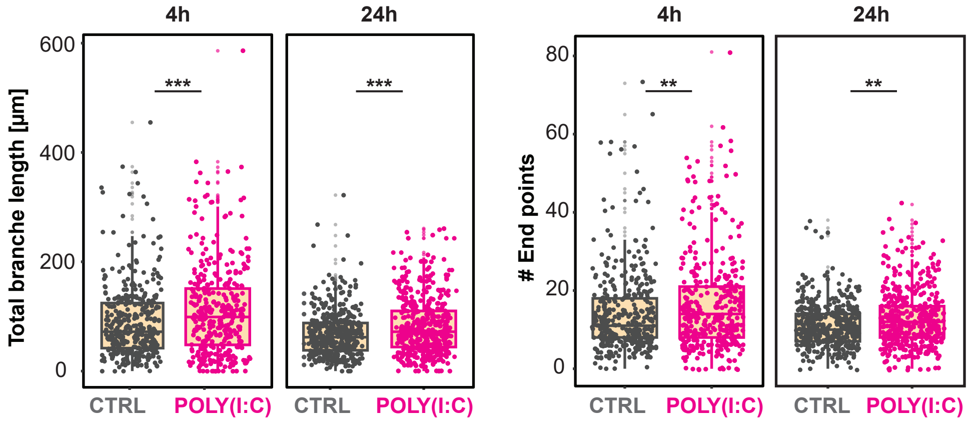

Supplementary Figure 7 – Timeline POLY(I:C)-mediated response.

a, Same assay as for in Supplementary Figure 6a with additional measurement of cytokine and chemokine release after two and four hours compared to 24h in iMG-$_{diss}$RO with annotated example membranes iMG-$_{diss}$RO.

b, Boxplot of the total branch length (left) and the number of endpoints (right) per iMG for CTRL (grey) and POLY(I:C) (magenta) following 4h and 24h stimulation in iMG-$_{diss}$RO. iMG were collected from five independent differentiations. Kruskal-Wallis test with post-hoc Dunn's test.

Supplementary Figure 8

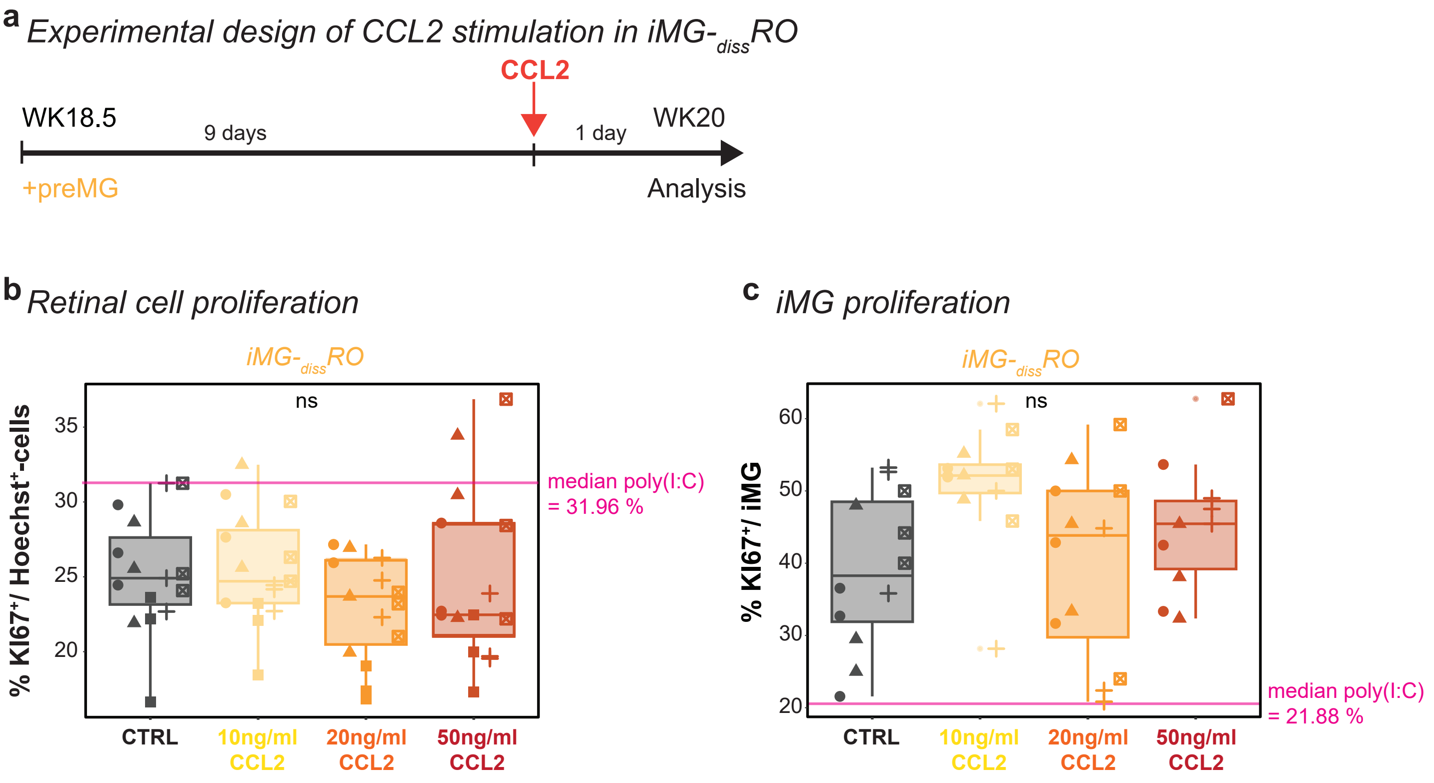

Supplementary Figure 8 – The POLY(I:C)-mediated proliferation rate increase cannot be replicated with CCL2 alone.

a, Experimental timeline. At WK18.5, preMG are added to $_{diss}$RO. After nine days, cultures received fresh medium for control (CTRL, grey) or CCL2 stimulation iMG-$_{diss}$RO for 24 hours.

b, Effect of CCL2 on retinal cell proliferation excluding iMG. Boxplot of percent KI67^{+}-cells relative to Hoechst^{+}-cells in iMG-$_{diss}$RO for CTRL and CCL2 stimulation at a final concentration of 10 ng/mL (yellow), 20 ng/mL (orange), and 50 ng/mL (red). Magenta line: Median proliferation rate in POLY(I:C) stimulation of iMG-$_{diss}$RO (**Figure 4g**). Symbols: three biological replicates from five independent differentiations. One-way ANOVA.

c, Effect of CCL2 on iMG proliferation. Boxplot of percent KI67^{+}/iMG for CTRL and CCL2 stimulation at a final concentration of 10 ng/mL (yellow), 20 ng/mL (orange), and 50 ng/mL (red). Magenta line: Median iMG-proliferation rate in POLY(I:C) stimulation of iMG-$_{diss}$RO (**Figure 4d**). Symbols: three biological replicates from five independent differentiations. Kruskal-Wallis test.

For detailed statistical analysis, see **Supplementary Table 4**.

*$p < 0.05$. $^{ns}p > 0.05$, not significant.

Supplementary Figure 9

a *Release of inflammatory mediators following ibuprofen treatment in iMG-$_{diss}$RO*

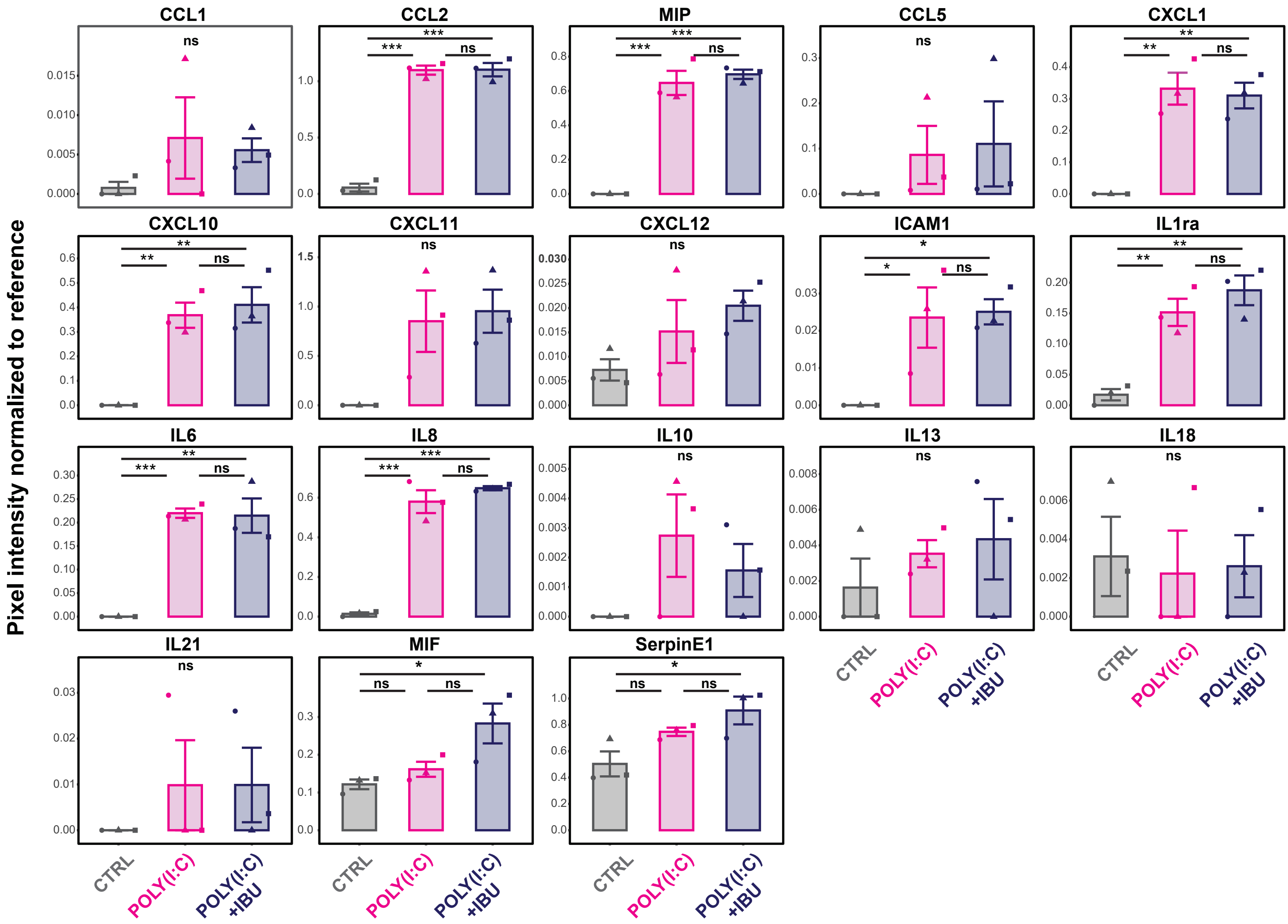

Supplementary Figure 9 –Comparison of individual secreted inflammatory mediators after ibuprofen exposure.

a, Release of inflammatory cytokines and chemokines into the supernatant based on the experimental paradigm described in **Figure 3a** for control (CTRL, grey), POLY(I:C) (magenta), and POLY(I:C) and S(+)-ibuprofen (POLY(I:C)+IBU, blue) stimulation. Release of different inflammatory mediators into the supernatant of iMG-$_{diss}$RO. Bar chart of pixel intensity normalized to reference with standard error of the mean for CTRL, POLY(I:C), and POLY(I:C)+IBU. Each symbol: an independent differentiation (n=3). One-way ANOVA with post-hoc Tukey's test, except IL13, IL18, IL21 Kruskal-Wallis test.

For detailed statistical analysis, see **Supplementary Table 4**.

***$p < 0.001$. **$p < 0.01$. *$p < 0.05$. $^{ns}p > 0.05$, not significant.

Supplementary Figure 10

Retinal cell types transduced with AAV2 7m/8

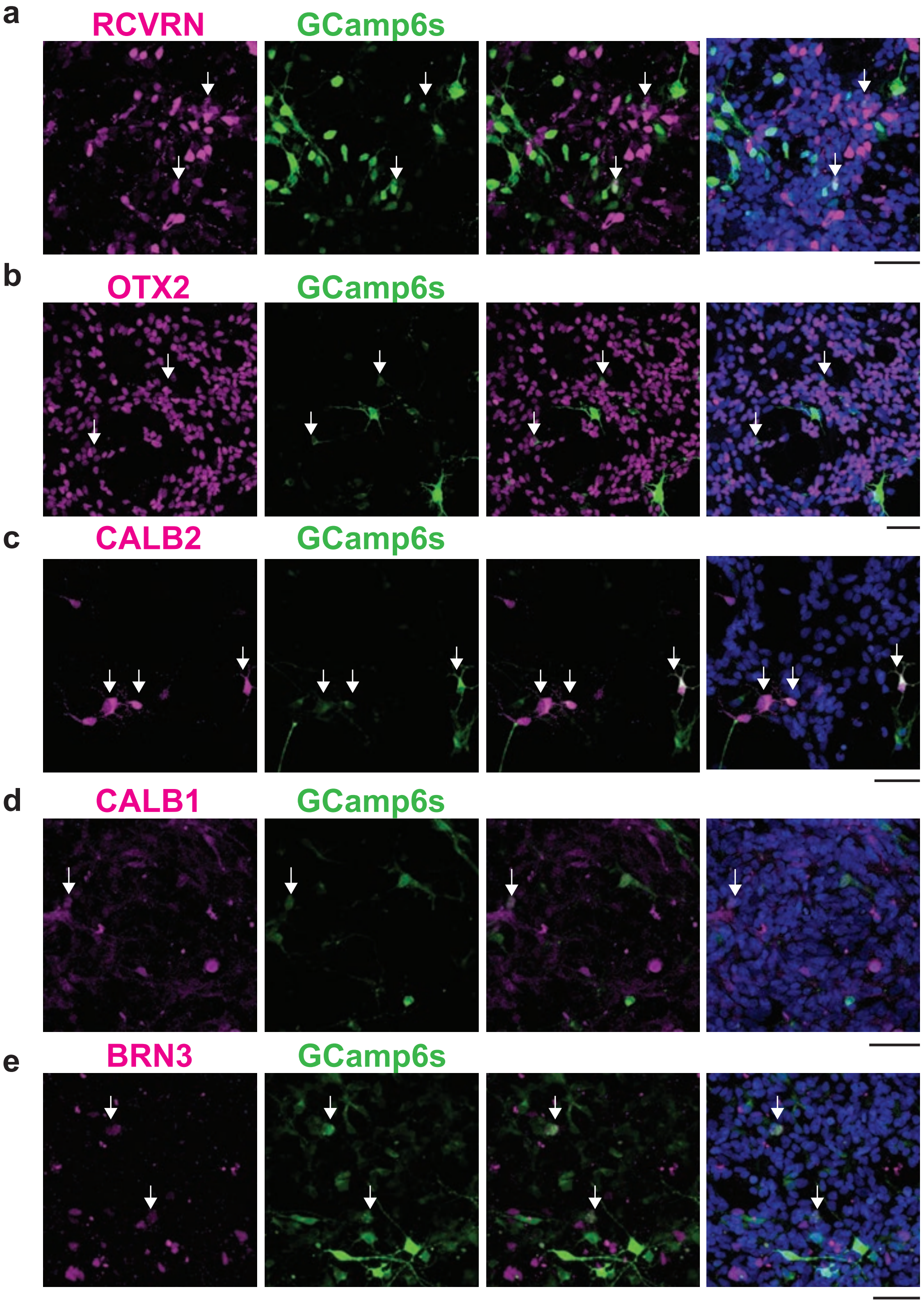

f *Calcium dynamics*

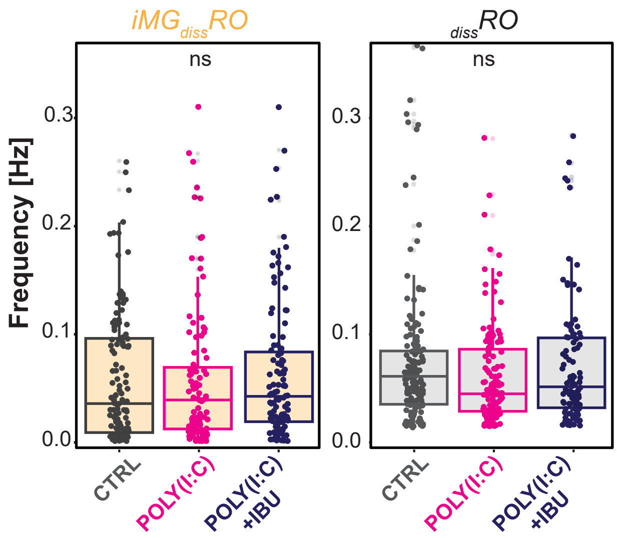

Supplementary Figure 10 –GCAMP6s expression across retinal cell types.
a-**e**, Example ROI images of $_{diss}$RO infected with AAV2-GCAMP6s at WK17, analyzed at WK20, counterstained for the nuclei-dye Hoechst (blue) and the calcium sensor GCAMP6s (green), and immunostaining for retinal cell types (magenta): **a**, RCVRN (recoverin; photoreceptors). **b**, OTX2 (orthodenticle homeobox 2; photoreceptors, bipolar cells). **c**, CALB2 (calretinin; photoreceptors, bipolar-, amacrine cells). **d**, CALB1 (calbindin; amacrine-, horizontal cells). **e**, BRN3 (brain-specific homeobox/POU domain protein 3B; ganglion cells). Arrow: Co-expression of calcium sensor and retinal marker. Scale bar: 50µm.
f, Spontaneous calcium dynamics in iMG-$_{diss}$RO (orange) and $_{diss}$RO (grey) for control (CTRL, grey), POLY(I:C) (magenta), and POLY(I:C) and S(+)-ibuprofen (POLY(I:C)+IBU, blue) stimulation. Boxplot of the mean frequency [Hz] during five minutes of recording. Each dot represents an active cell. Recordings from five biological replicates from independent differentiations. Kruskal-Wallis test.

For detailed statistical analysis, see **Supplementary Table 4**.
$^{ns}p > 0.05$, not significant.

Supplementary Figure 11

a *Expression in UCSC Cell Browser of Cowan et al., 2020*

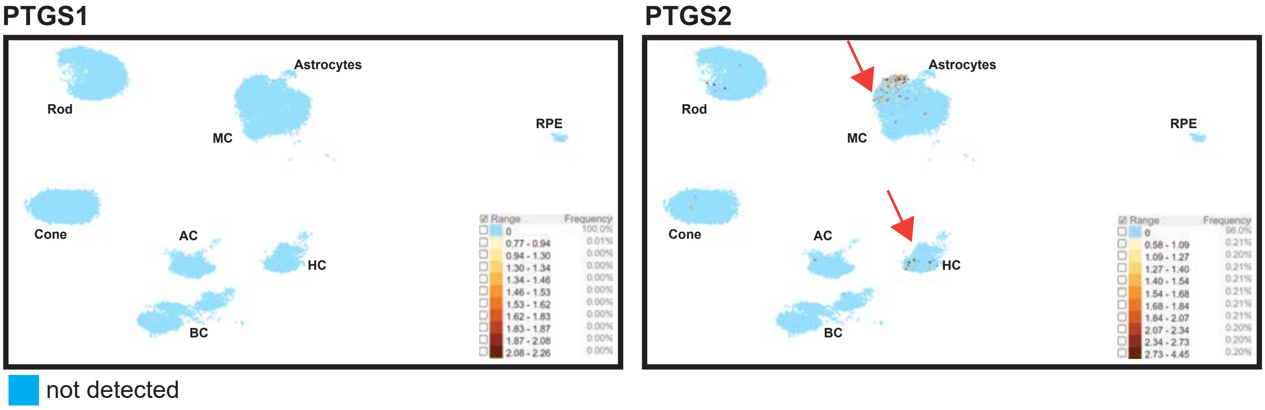

b *Expression of TLR3 in UCSC Cell Browser of Cowan et al., 2020*

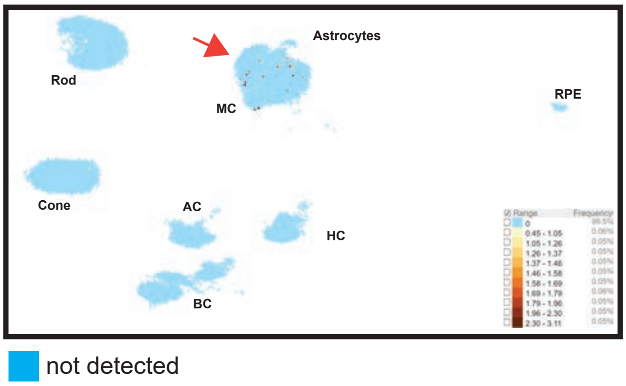

c *hIPSC-derived astrocytes*

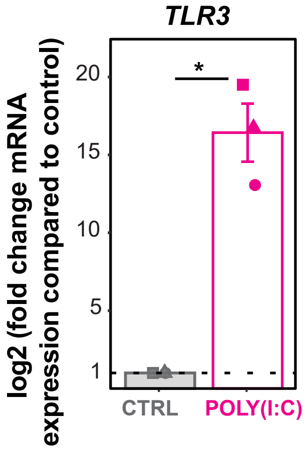

Supplementary Figure 11 – PTGS1, PTGS2, and TLR3 mRNA expression profile.
a-**b**, Expression of (**a**) PTGS1 and PTGS2 (prostaglandin-endoperoxide synthase 1 and 2) as well as (**b**) TLR3 (toll-like receptor 3) in USCS Cell Browser of *Cowan et al., 2020*. Cell Browser dataset ID: 'Developed human retinal organoid.' Uniform manifold approximation and projection (UMAP) of transcript expression. AC: amacrine cell. BC: bipolar cell. Cone: cone photoreceptors. HC: horizontal cell. MC: Müller glia. RPE: retinal pigment epithelium. Rod: rod photoreceptors. Red arrow: positive transcript expression. Blue dot: not detected.
c, Real-time quantitative polymerase chain reaction (RT-qPCR) for TLR3 (toll-like receptor 3) in hIPSC-derived astrocytes for untreated control (CTRL, grey) and POLY(I:C) (magenta) exposure. Mean mRNA transcript log2-fold changes compared to untreated control cells with standard error of the mean. Each symbol is an independent differentiation (n=3). One sample t-test.

For detailed statistical analysis, see **Supplementary Table 4**.
*$p < 0.05$.
