# Supplementary material for: Microglia determine an immune-challenged environment and facilitate ibuprofen action in human retinal organoids
Source: J Neuroinflammation. 2025 Apr 3;22:98. doi: 10.1186/s12974-025-03366-x (PMC11966913; doi:10.1186/s12974-025-03366-x)
Supplement: Supplementary file 2 — Supplementary Material 2. Table 1. Overview of human induced pluripotent stem lines (hIPSC) included in this study. [file 12974_2025_3366_MOESM2_ESM.docx]

| **Cell line** | **SC 102A-1** | **01F49i-N-B7** |
| --- | --- | --- |
| **hPSCreg.eu** | SBLi006-A |  |
| **Company** | BioCat |  |
| **Generator** | SYSTEM BIOSCIENCES | Institute of Molecular and Clinical Ophthalmology Basel; Cowan et al 2020, Cell |
| **Abbreviation within manuscript** | SC102A | F49B7 |
| **Source** | Fibroblast (dermis) | Fibroblast |
| **Vector** | Retrovirus (integrating) | Sendai virus |
| **Genes for reprogramming** | MYC, KLF4, SOX2, OCT4/POU5F1 | Oct3/4, Sox2, Klf4, and cMyc |
| **Health status** | Healthy | unknown |
| **Race** | Caucasian | unknown |
| **Sex** | Male | Female |
| **Donor age** | 60-64 |  |

**Supplementary Table 1**

hPSCreg.eu, human pluripotent stem cell registry. MYC, MYC proto-oncogene. KLF4, kruppel-like factor 4. Large T antigen, large tumor antigen. LIN28, zinc finger CCHC domain-containing protein. OCT4 (octamer-Binding Protein 4)/ POU5F1 (POU domain, class 5, transcription factor 1). SOX2, sex-determining region Y-box 2. SV40, simian-virus 40.
