# Supplementary material for: Microglia determine an immune-challenged environment and facilitate ibuprofen action in human retinal organoids
Source: J Neuroinflammation. 2025 Apr 3;22:98. doi: 10.1186/s12974-025-03366-x (PMC11966913; doi:10.1186/s12974-025-03366-x)
Supplement: Supplementary file 3 — Supplementary Material 3. Table 2. List of antibodies. [file 12974_2025_3366_MOESM3_ESM.docx]

**Supplementary Table 2 – Antibodies**

| **Antibody** | **Host** | **Vendor** | **Catalogue #** | **Lot #** | **Dilution factor** | **RRID number** |
| --- | --- | --- | --- | --- | --- | --- |
| **BRN3B** | Goat | Santa Cruz Biotechnology | sc-6026 | K0215 | 100 | AB_673441 |
| **Cleaved Caspase3** | Rabbit | Cell Signaling Technology | 9661 | 47 | 100 | AB_2341188 |
| **CALB2** | Mouse | Swant | 6B3 |  | 100 | AB_10000320 |
| **CALB1** | Guinea pig | Synaptic Systems | 214 004 | 1-15 | 200 | AB_10550535 |
| **CD45** | Rabbit | Cell Signaling Technology | 13917P | 1 | 200 | AB_2750898 |
| **ChAT** | Goat | EMD Millipore | AB144P | 3182642 | 400 | AB_2079751 |
| **CX3CR1** | Mouse | BioLegend | B355702 | B194773 | 50 | AB_2561726 |
| **GFAP** | Rat | STEMCELL Technologies | 60048.1 | 1000079097 | 100 | AB_3095092 |
| **IBA1** | Rabbit | GeneTex | GTX100042 | 44200 | 750 | AB_1240434 |
| **IBA1** | Goat | Abcam | ab5076 | GR3374909-1 | 250 | AB_2224402 |
| **ITGAM** | Chicken | Acris Antibodies | AP31807PU-N | MAC7967984 | 100 | AB_11146887 |
| **KI67** | Mouse | BD Biosciences | 550609 | 5267542 | 100 | AB_393778 |
| **MAP2** | Chicken | EMD Millipore | AB5543 | 4045792 | 250 | AB_571049 |
| **myb** | Rabbit | Acris | AP31223PU-N | 27931 | 100 | AB_10976997 |
| **NANOG** | Rabbit | Proteintech/ THP medical product | #14295-1-AP | 00019675 | 200 | AB_1607719 |
| **OCT3/4** | Mouse | BD Biosciences | #611202 | 4052889 | 100 | AB_398736 |
| **OTX2** | Goat | R&D Systems | AF1979 | KNO0920111 | 150 | AB_2157172 |
| **P2Y12** | Rabbit | Sigma-Aldrich | HPA014518 | F119293 | 100 | AB_2669027 |
| **PRKCA** | Mouse | BD Biosciences | 610107 | K1315 | 100 | AB_397513 |
| **PSD95** | Rabbit | Cell Signaling Technology | 3450 | 5 | 200 | AB_2292883 |
| **PU.1** | Rabbit | Cell Signaling Technology | 2266S | 1 | 500 | AB_10692379 |
| **RCRVN** | Rabbit | EMD Millipore | AB5585 | 2691407 | 400 | AB_2253622 |
| **RIBEYE** | Mouse | EMD Millipore | MABN804 | Q2583290 | 100 | AB_3271577 |
| **RLBP1** | Mouse | Abcam | ab15051 | GR229880-2 | 200 | AB_2269474 |
| **RUNX.1** | Mouse | BioLegend | 659302 | B276756 | 50 | AB_2563194 |
| **TREM2** | Goat | R&D Systems | AF1828 | JWF0719111 | 100 | AB_2208689 |
| **VGLUT1** | Guinea pig | Synaptic Systems | 135304 | 4-73 | 100 | AB_887878 |
