# Supplementary material for: Microglia determine an immune-challenged environment and facilitate ibuprofen action in human retinal organoids
Source: J Neuroinflammation. 2025 Apr 3;22:98. doi: 10.1186/s12974-025-03366-x (PMC11966913; doi:10.1186/s12974-025-03366-x)
Supplement: Supplementary file 4 — Supplementary Material 4. Table 3. Primer sequences. [file 12974_2025_3366_MOESM4_ESM.docx]

**Supplementary Table 3 – Primer sequences**

| **Target** | **Gene** | **Primer pair** | **Primer sequence 5'-3'** |
| --- | --- | --- | --- |
| House keeping genes | OAZ1 | Forward | AGGACAGCTTTGCAGTTCTC |
|  |  | Reverse | CGGTTCTTGTGGAAGCAAATG |
|  | GAPDH | Forward | GTCTCCTCTGACTTCAACAGCG |
|  |  | Reverse | ACCACCCTGTTGCTGTAGCCAA |
|  | ACTB | Forward | CACCATTGGCAATGAGCGGTTC |
|  |  | Reverse | AGGTCTTTGCGGATGTCCACGT |
|  | RPL27 | Forward | ATCGCCAAGAGATCAAAGATAA |
|  |  | Reverse | TCTGAAGACATCCTTATTGACG |
| Gene of interest | PTGS1 | Forward | GATGAGCAGCTTTTCCAGACGAC |
|  |  | Reverse | AACTGGACACCGAACAGCAGCT |
|  | PTGS2 | Forward | CCCTTCTGCCTGACACCTTT |
|  |  | Reverse | TTCTGTACTGCGGGTGGAAC |
|  | TLR3 | Forward | CCTTTTGCCCTTTGGGATGC |
|  |  | Reverse | TGAAGTTGGCGGCTGGTAAT |
|  | C1Qa | Forward | GTGACACATGCTCTAAGAAG |
|  |  | Reverse | GACTCTTAAGCACTGGATTG |
|  | CX3CR1 | Forward | CTTACGATGGCACCCAGTGA |
|  |  | Reverse | CAAGGCAGTCCAGGAGAGTT |
|  | P2RY12 | Forward | GATGCCACTCTGCAGGTTG |
|  |  | Reverse | GTGCACAGACTGGTGTTACC |
|  | TMEM119 | Forward | CACGGACTCTCTCTTCCAG |
|  |  | Reverse | GCAGCAACAGAAGGATGAGG |
